# Elucidating the Microscale Behavior and Phase Separation Kinetics of Thermally Responsive Ionic Liquid–Water Mixtures

**DOI:** 10.1021/acsami.5c24522

**Published:** 2026-02-12

**Authors:** Ahmed Mahfouz, Jordan D. Kocher, Andrew Z. Haddad, Akanksha K. Menon

**Affiliations:** † George W. Woodruff School of Mechanical Engineering, 1372 Georgia Institute of Technology, Atlanta, Georgia 30332, United States; ‡ Energy Storage and Distributed Resources Division, 1666 Lawrence Berkeley National Laboratory, Berkeley, California 94720, United States

**Keywords:** thermally responsive ILs, LCST, phase separation, colloidal aggregates, kinetics, desalination

## Abstract

Thermally responsive ionic liquids (ILs) exhibit liquid–liquid phase separation into a water-rich (WR) and ionic-liquid-rich (ILR) phase when heated above a lower critical solution temperature (LCST). This phase behavior has been leveraged for applications ranging from forward osmosis (FO) desalination, where the IL acts as a draw solute, to refrigeration and dehumidification cycles, where the IL acts as a liquid desiccant. While significant effort has been devoted to characterizing the thermodynamic and thermophysical properties of LCST ILs, their phase separation kinetics have not been investigated. In this work, we describe the macroscale phase separation kinetics (phase separation time) by gleaning insight into the microscale colloidal behavior of aqueous mixtures of four different materials, P_4444_TFA (tetrabutylphosphonium-2,4-trifluoroacetate), P_4444_DMBS (tetrabutylphosphonium-2,4-dimethyl-benzenesulfonate), N_4444_Sal (tetrabutylammonium salicylate), and P_4444_Sal (tetrabutylphosphonium salicylate) as a function of IL concentration at a separation temperature of 70 °C. We report the discontinuous microscale size distributions for each material and correlate their theoretical settling velocities to experimental phase separation times. The results indicate that a simple Stokes’ law model can predict the phase separation time within reasonable accuracy. Overall, this work lays the foundation for understanding the micro- to macroscale phase separation behavior and kinetics of LCST ILs for various water-energy applications.

## Graphical abstract

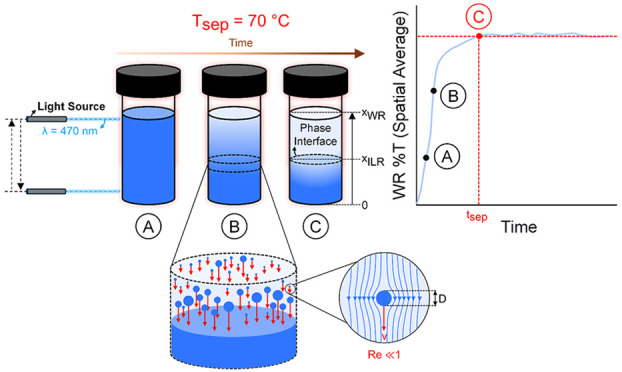

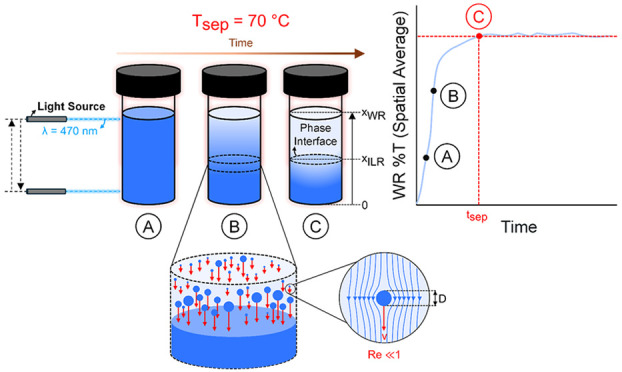

## Introduction

Room temperature ionic liquids (ILs) are an emerging class of materials that have been employed in a variety of applications, including protein extraction,^1,2^ lithium^1,3^ and uranium^1,3^ recovery from seawater, desalination,^3–5^ and carbon capture.^3,4,6^ These ILs are characterized by a low melting point <100 °C, high chemical and thermal stability, negligible vapor pressure, and high ionic conductivity.^7^ The stability and low volatility of ILs arise from the steric hindrance between the ions in solution, which is much greater than van der Waals forces and hydrogen bonding in molecular liquids.^8^ In recent years, a class of ionic liquids (ILs) that exhibits thermally responsive phase behavior when mixed with water has emerged as a working fluid for applications ranging from desalination^9–11^ and solvent extraction,^12^ to dehumidification and refrigeration^13–15^ and atmospheric water harvesting.^16^ First explored by Ohno and co-workers in 2013, these thermoresponsive ILs are typically comprised of a hydrophilic cation and a hydrophobic anion.^17,18^ These IL-water mixtures exhibit liquid–liquid phase separation when heated above a lower critical solution temperature (LCST), separating into a water-rich (WR) phase and an IL-rich (ILR) phase.^8,10,12,17–23^ This thermoresponsive behavior emerges as a consequence of a finely tuned balance of the hydrophobic and hydrophilic moieties that comprise these ILs.^10,24^

For a given concentration of IL in aqueous solution (*w*_IL_) at an initial temperature *T*_i_ < *T*_C_ (critical temperature), the IL is fully miscible in water and forms a homogeneous single-phase solution, as illustrated in Figure 1A. Upon heating to a separation temperature *T*_sep_ > *T*_C_, the solution separates into a WR and ILR phase. The low enthalpy of separation (∼460× lower than the enthalpy of vaporization of water) and phase separation temperatures (<60 °C) have generated significant interest in using these materials.^9,11,13,15,18,20,21,25–30^ In such applications, LCST ILs are used as a draw solution or a desiccant, absorbing and desorbing only liquid water using a selective membrane in the case of forward osmosis (FO) desalination^9–11,20,28,9–11,30–33^ or water vapor from moist air in the case of dehumidification and/or refrigeration.^13,29^ The saturated draw/desiccant is then heated to a temperature above *T*_C_ to regenerate the WR and ILR phases; the WR phase is subsequently purified to remove residual IL content (*w*_IL,WR_ in Figure 1A) in the case of FO desalination,^9^ or evaporated to the ambient environment to induce evaporative cooling in the case of refrigeration.^13^

**Figure 1:**
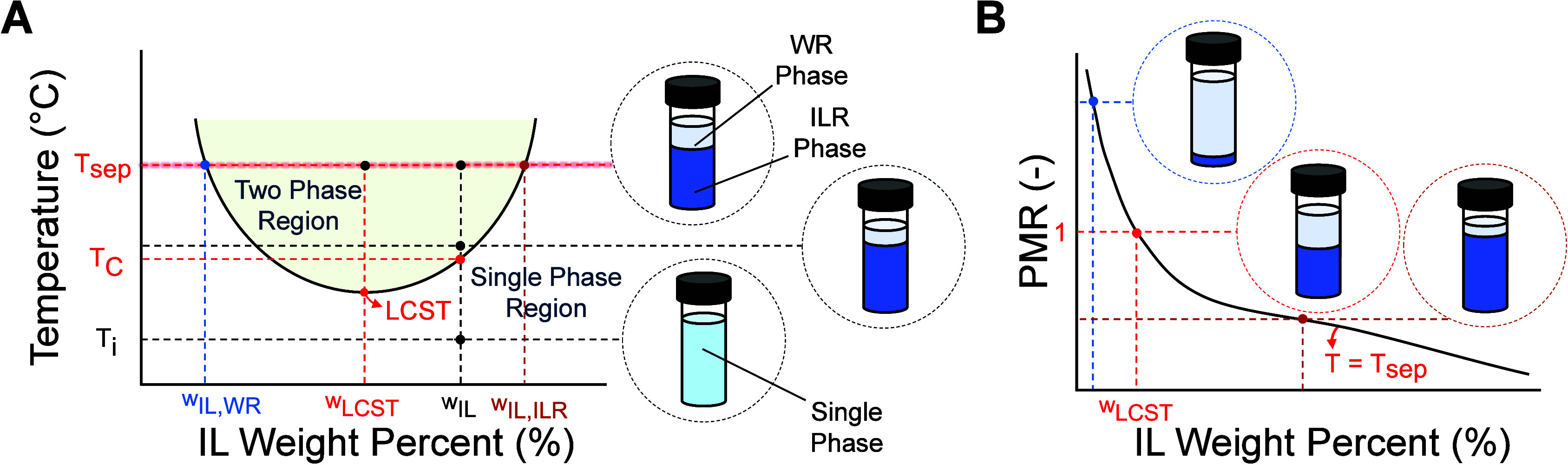
Overview of thermally responsive ionic liquids. (A) Binodal phase diagram showing the LCST behavior of IL–water mixtures. (B) Phase mass ratio (PMR) of IL–water mixtures as a function of concentration at *T*_sep_. Figure 1A is reproduced from Mahfouz et al.^28^ Available under a CC-BY 3.0 license. Copyright 2024 Mahfouz et al.

The purity of both phases and the WR to ILR phase mass ratio (PMR) increase with *T*_sep_, as illustrated in Figure 1A. At a given *T*_sep_, the PMR declines monotonically with increasing IL concentration, as illustrated in Figure 1B, as a consequence of the phase diagram tie rule.^28^ This trend in the PMR has been experimentally characterized for some thermally responsive ILs.^28^ The LCST and PMR depend on the hydrophilicity of the cation and the hydrophobicity of the anion: the more hydrophilic the cation, the higher the LCST,^34^ and the more hydrophobic the anion, the lower the PMR (less mass of the WR phase is formed at a given *T*_sep_). A low LCST reduces the temperature of the heat needed to induce phase separation, while a higher PMR enables the regeneration of more mass of the WR phase.^9,31^ These two parameters (LCST and PMR) are thus not only important from an application standpoint, but they also pave the way for understanding the microscale phase separation of IL-water mixtures, as we show in this work.

The fundamental mechanism that drives LCST phase separation is the formation of micelle-like aggregates of water molecules and IL ions in solution.^10,35^ This induces ordering that results in a negative entropy of mixing, Δ*S*_mix_, while the enthalpy of mixing, Δ*H*_mix_, is negative due to the cohesive interactions between the water and IL.^36^ For a given *w*_IL_, at temperatures below *T*_C_, the Gibbs free energy of mixing (Δ*G*_mix_ = Δ*H*_mix_ – *T*Δ*S*_mix_), is negative and IL-water forms a single phase spontaneously. Upon heating the solution to a separation temperature *T*_sep_ ≥ *T*_C_, Δ*G*_mix_ becomes positive, indicating unfavorable mixing or two separate phases. For LCST materials, spinodal decomposition results in the formation of two microscopically distinct phases that ultimately coalesce into macroscopically distinct immiscible phases.^37–40^ Spinodal decomposition is different from other phase separation processes, such as nucleation and growth, in that there is no nucleation barrier, resulting in a nearly instantaneous formation of uniformly dispersed microscopically distinct phases.^41,42^

To better understand this, the nonideal interactions between IL and water occurring at the microto-macroscale must be considered. It has been shown that (i) the WR and/or ILR phase form long-living discontinuous monodispersed aggregates of 10–100 μm; the size depends on temperature, IL concentration, and IL species.^21,43^ This colloidal behavior has also been reported for deep eutectic solvents^44,45^ and hydrogels^46^ that exhibit LCST. Additionally, (ii) beyond a critical IL concentration, the surface tension of IL-water mixtures approaches a constant value due to the formation of micellar aggregates in solution. These micellar formations have been imaged in IL water mixtures by Gao et al.,^47^ however, critical micelle concentration (CMC) is exhibited only by aqueous ILs that possess an LCST.^10^ Finally, (iii) the osmolality (water activity) remains nearly constant for IL concentrations between ∼10–50 wt %, suggesting the formation of micellar aggregates that reduce the number of free ions in solution.^9–11,28,31^

Whereas studies in existing literature have focused exclusively on the thermodynamics of LCST ILs (osmotic strength and critical temperatures), their phase separation kinetics have not been systematically investigated, which is of critical importance from an application standpoint.^29^ Furthermore, in a real process, the separation kinetics directly impact the purity of the WR and ILR phases.^28^ This requires an understanding of the microscale behavior of LCST ILs, which is currently lacking, with only two literature studies over the past decade.^21,43^

To address this gap, this work characterizes the microscale behavior of four LCST ILs at different concentrations at *T*_sep_ = 70 °C using optical microscopy, which, to our knowledge, has not been directly reported in existing literature. This information is then used to determine the macroscopic phase separation time required for each material at each concentration, and is compared with the experimental settling time of the phasic colloidal aggregates. These findings provide seminal insight into the microscale phase separation process of LCST ILs and comprehensively bridge the gap between micro and macro scale phasic development. Our findings highlight the importance of accounting for the time scales associated with the phase separation process of LCST ILs, which can be a major limiting factor for industrial processes.

## Experimental Methods

### Materials and Synthesis

The four ILs studied in this work are shown in Figure 2. The cations are tetrabutylammonium ([N_4444_]^+^) and tetrabutylphosphonium ([P_4444_]^+^); the anions are salicylate ([Salicyl]−), 2,4-dimethylbenzene-sulfonate ([DMBS]^−^), and trifluoroacetate ([CF_3_COO]^−^). For simplicity, the ILs are designated as follows: [N_4444_]^+^ [Salicyl]^−^ as “NSal”, [P_4444_]^+^ [CF_3_COO]^−^ as “PTFA”, [P_4444_]^+^ [Salicyl]^−^ as “PSal”, and [P_4444_]^+^ [DMBS]^−^ as “PDMBS”. All ILs are solid at room temperature, with a melting point around 60–80 °C. These ILs are chosen as they have been reported in literature for various water-energy applications.^9,10,16,20,23,28–30^

**Figure 2:**
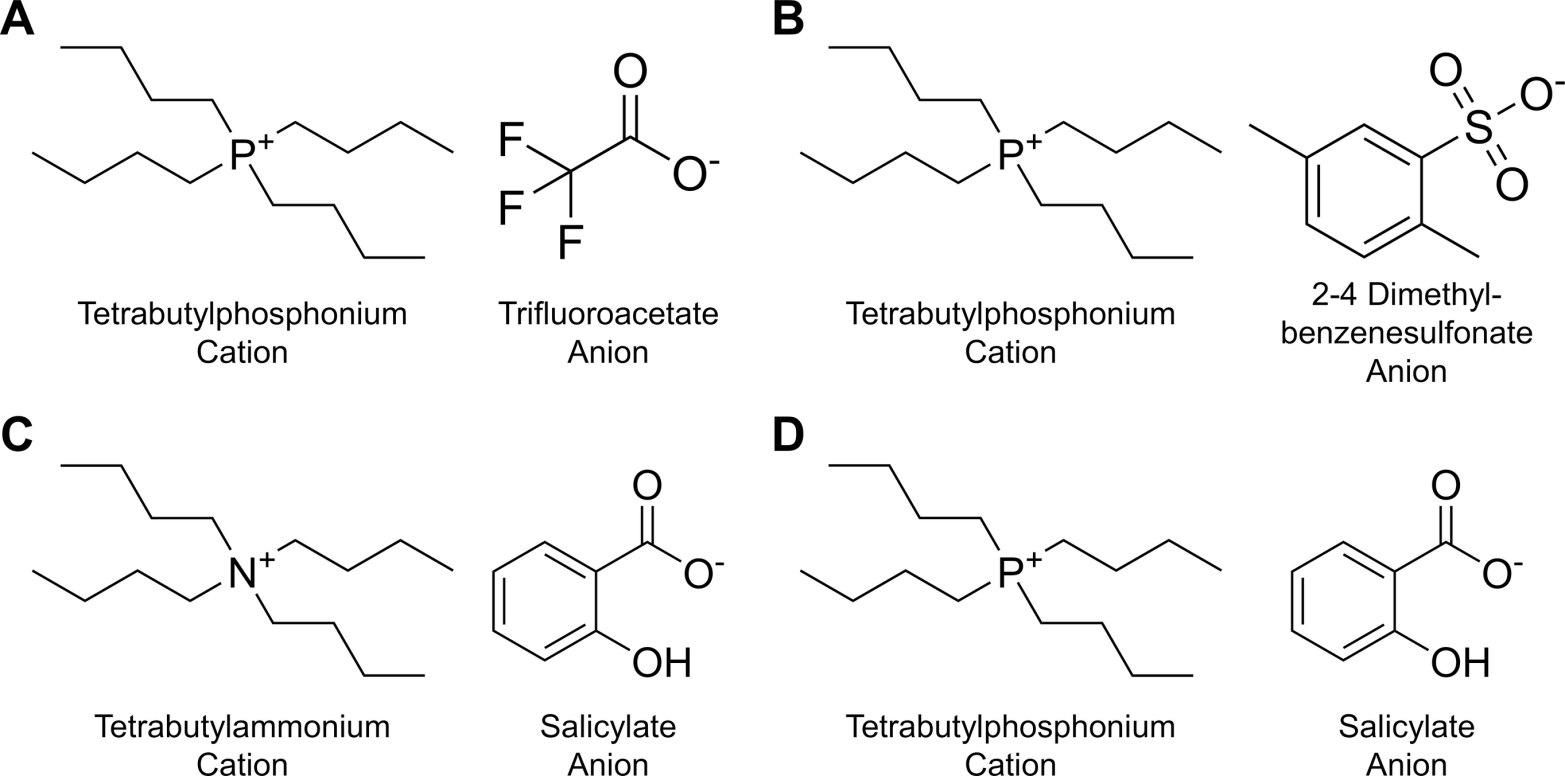
Chemical structures of the ILs studied in this work and their molar mass (*M*). (A) PTFA (*M*_PTFA_ = 372.02 g mol^–1^). (B) PDMBS (*M*_PDMBS_ = 458.25 g mol^–1^). (C) NSal (*M*_NSal_ = 364.11 g mol^–1^). (D) PSal (*M*_PSal_ = 396.54 g mol^–1^).

PTFA, PDMBS, NSal, and PSal were synthesized following the procedure outlined in our previous work.^28^ FTIR and NMR were performed to confirm the synthesis of the four ILs and their purity.

### Characterization Methods

Deionized water was used to prepare all aqueous IL mixtures. A mass balance with a resolution of 0.001 g (A&D FX-300i) was used to prepare mixtures over a range of IL concentrations.

#### Optical Microscopy for Size Distribution

The microscale size distribution for a mixture at a given concentration was characterized using an optical microscope (Keyence VHX-S650E light microscope with the ZS-20 lens) as illustrated in Figure 3A. To visualize the differences between the WR and ILR phases, 0.1 g of Nile blue sulfate (N5632 Sigma-Aldrich) was dissolved in a 3 mL IL-water mixture. A 30 μL sample is then pipetted onto a microscope slide (VWR VistaVision, 3 in × 1 in × 1 mm) and sandwiched with another slide to prevent evaporation, resulting in a ∼300–400 μm film. The sample was placed on a ceramic hot plate (VWR) set at 70 °C to induce phase separation by heating for 5 min to reach thermal equilibrium with the hot plate surface before imaging. Different illumination options (coaxial, ring, or a mix of the two) were utilized for each sample to obtain high-quality images of the aggregates. The images are then analyzed using the open-source ImageJ software to calculate the size distributions.

**Figure 3:**
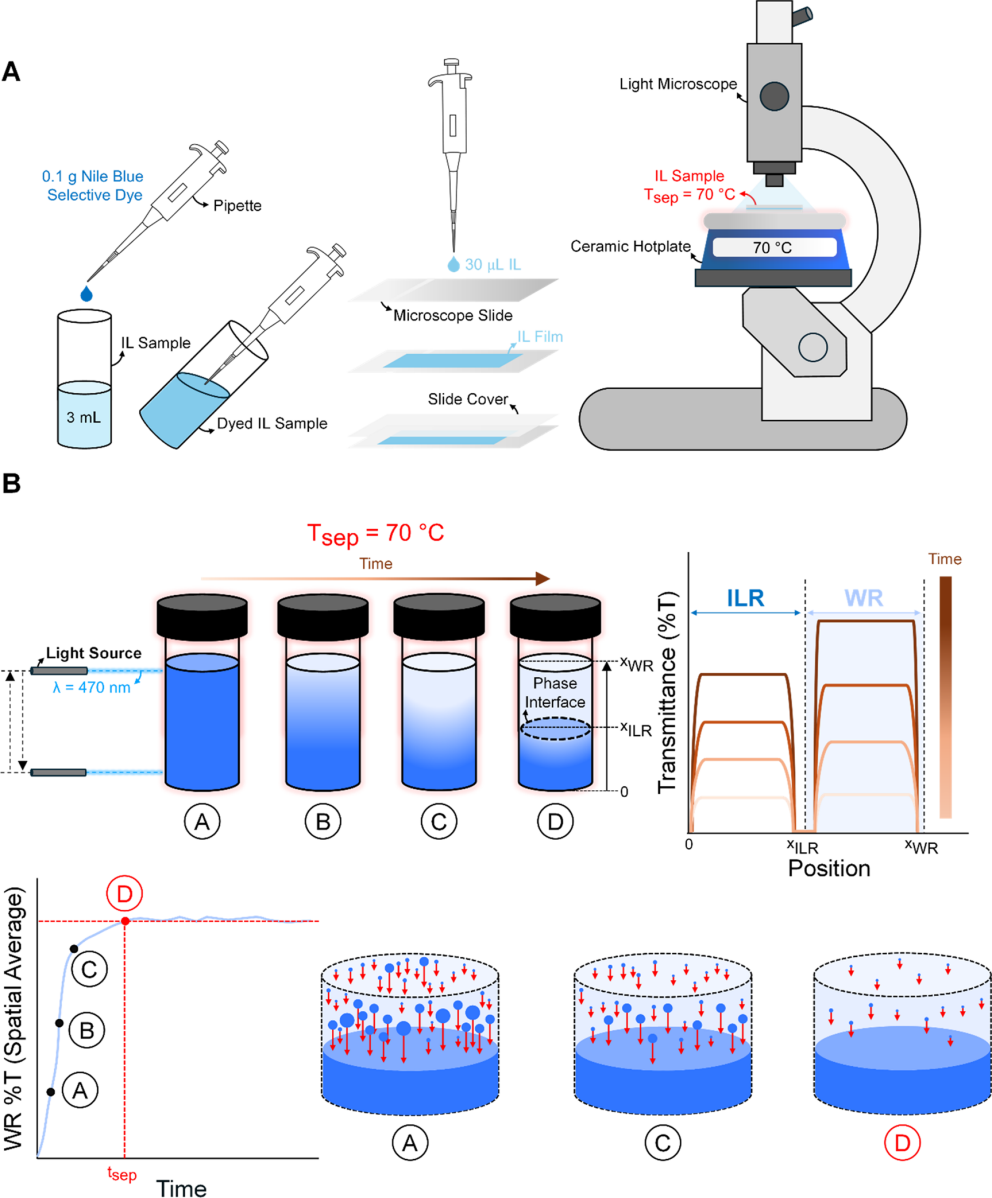
Illustration of experimental procedures used in this work to characterize the phase separation kinetics of IL-water mixtures. (A) Microscopic imaging procedure. 0.1 g of Nile blue-colored selective dye is added to a 3 mL IL-water solution. The selective dye dissolves in the IL content of the solution, creating a clear color contrast between the WR and ILR phases. A 30 μL droplet is then transferred to a microscope slide, creating a uniform film as illustrated. The microscope slide is then placed on a ceramic hot plate set to 70 °C for imaging. (B) Position variable transmittance measurement technique used to determine macroscopic phase separation time, *t*_sep_.

#### Light Transmittance for Phase Separation Time

At the onset of phase separation, an LCST IL will turn cloudy and gradually become transparent as phase separation progresses.^28,48^ To characterize the time required for macroscopic phase separation, a position-variable light transmittance measurement technique was used (DataPhysics MultiScan MS20). For this, a 10 mL sample (without the Nile blue sulfate dye) was prepared in a cylindrical glass vial. This instrument records the transmittance of the sample (along the height of the glass vial) at a wavelength of 470 nm at a spatially uniform temperature of *T*_sep_ = 70 °C, as illustrated in Figure 3B. These spatial transmittance profiles, when averaged over the WR phase, approach a constant transmittance over time, indicating complete macroscopic phase separation. Position-variable light transmittance measurements were performed at a spatial resolution of 0.055 mm and a temporal resolution of 10 s; the maximum repeatability error of the 470 nm light source is ±0.05% of the measured transmittance at a given point. For each sample, the maximum height *x*_WR_ was set to 2 cm as illustrated in Figure 3B. Due to the meniscus that forms at the interface of the WR and ILR phases from their surface tension differences, the transmittance at *x*_ILR_ is below 1–5% at all times due to light scattering across all IL samples.^10^

To enable a comparison between this experimentally measured macroscopic phase separation time and the Stokes’ settling time model of the mean colloidal size (*d̅*), the experimental phase separation time (*t*_sep_) was set to correspond to 90% of the maximum transmittance. Given that the size distributions of all mixtures at a given concentration are found to be Gaussian and nearly monodispersed (peaking around the mean), this transmittance value corresponds to the settling of the bulk of the colloidal population (approximately greater than μ – 1.6σ, where μ and σ are the mean and standard deviation, respectively, of a Gaussian distribution), as illustrated in Figure 3B and discussed further in the Results section. The measurement was repeated three times for each sample at H* = 2 cm at a given concentration to determine the standard deviation associated with the *t*_sep_ value. Detailed error propagation analysis from the experimental measurables (*H*, *d̅*, μ_*c*_, and Δρ) was performed to determine the theoretical uncertainty bands in theoretical phase separation time calculations (Supporting Note 7).

#### Phase Density

A density meter (Mettler Toledo Hand-held Densito) was used to characterize the density of the WR and ILR phases for the four ILs at *T*_sep_ = 70 °C. For this, 10 g of a 40 wt % mixture (*x*_WR_ = 3 cm) was prepared for each IL and heated in a water bath at 70 °C. The separated phases were then pipetted into different vials and allowed to cool down to 50 °C (temperature limit of the density meter) as illustrated in Figure S1A. The density of each phase was measured three times to determine the mean phase density difference, Δρ = ρ_ILR_ – ρ_WR_, as shown in Figure S2. The density meter has a resolution of 0.0001 g/cm^3^ and an accuracy of ± 0.001 g/cm^3^.

#### Phase Viscosity

A viscometer (RheoSense m-VROC) was utilized to measure the viscosity of the WR and ILR phases of the four ILs at *T*_sep_ = 70 °C at a constant shear rate of 1000 s^–1^ over 2 min, as illustrated in Figure S1B. Given that the ILs exhibit Newtonian behavior, the dynamic viscosity is independent of the shear rate.^9,10^ The viscosity measurements are reported in Figure S3. The temperature control accuracy is ±1 °C, with a viscosity accuracy/repeatability of ±2%.

## Results and Discussion

This section discusses the microscale behavior of IL-water mixtures and how it influences the macroscale LCST phase separation kinetics. We first discuss the relevance of the binodal phase diagrams and the PMR trends for the four ILs.^28^ Then we discuss their microscale colloidal behavior at *T*_sep_ = 70 °C for concentrations between 10–50 wt %. Finally, we report the macroscopic phase separation time, *t*_sep_, for each mixture in the same concentration range, and then compare these experimental results to Stokes’ law of settling.

### Elucidating the Microscale Colloidal Phase Behavior of LCST ILs

The microscale behavior of thermally responsive IL-water mixtures is summarized in Figure 4. The results show that PMR at a given temperature (Figure 1B) is the key parameter that governs the microscale behavior of LCST ILs. Specifically, at high PMR values >2.75 (i.e., mass of the WR phase is ∼2.75× that of the ILR phase), the ILR phase forms discontinuous aggregates within a continuous WR phase. Since these ILs are denser than water,^10,28^ the ILR discontinuous aggregates are denser than the WR phase and gradually settle below the WR phase, forming two macroscopically distinct phases over time. On the other hand, at low PMR values <0.75, the aggregates exhibit complete phase inversion, with discontinuous WR phase aggregates forming within a continuous ILR phase, which rise due to their lower density. This behavior is universal, as it is observed in all four ILs studied in this work.

**Figure 4:**
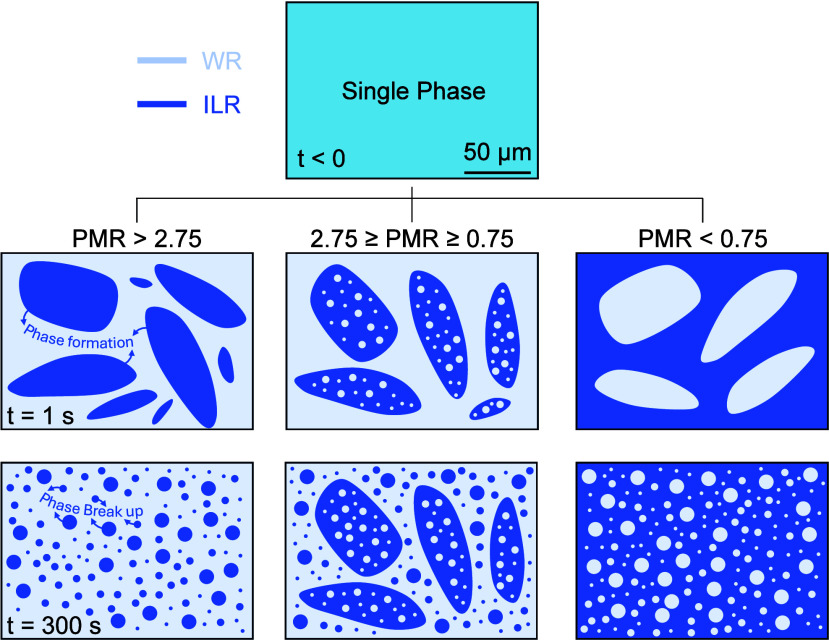
Illustration of the universal microscale colloidal behavior observed for LCST ILs at *T*_sep_ = 70 °C over time. At PMR > 2.75, the ILR phase forms discontinuous colloidal aggregates within a continuous WR phase. At 2.75 ≥ PMR ≥ 0.75, both the WR and ILR phases form discontinuous colloidal aggregates. At PMR < 0.75, the WR phase is discontinuous within a continuous ILR phase. The ILR phase is shown in a darker color, and the WR phase is shown in a lighter color.

At intermediate PMR values between ∼0.75–2.75, both the WR and ILR phases form discontinuous aggregates, as illustrated in Figure 4. This behavior is observed for three of the ILs except PTFA, which is consistent with the findings of Wang et al. For PTFA, the microscale behavior is such that phase inversion is observed between the low and high PMR without an intermediate regime in which both phases form discontinuous aggregates.^43^ We hypothesize that this atypical behavior exhibited by PTFA is attributed to the highly basic nature TFA anion,^49,50^ whereas the DMBS and Sal anions are both acidic.^51–53^ Since the solubility of an IL in water is inversely proportional to the basicity of the anion,^54,55^ the ILR phase of PTFA is more likely to form discontinuous aggregates within the WR phase compared to the other three ILs.

Following the experimental procedure illustrated in Figure 3A, the aggregate size distribution is determined at *T*_sep_ = 70 °C for PTFA, PDMBS, NSal, and PSal at IL concentrations between 10 and 50 wt %. This is shown as a function of concentration for PTFA and NSal in Figures 5 and 6 to highlight the evolution of the phase behavior. If both phases form discontinuous aggregates, the size distribution of the phase with the highest population of aggregates is reported. Additional microscopic images, including for PDMBS and PSal, and the size distribution of the aggregates of the discontinuous phase are shown in Figures S10–S13. For all four LCST ILs at all concentrations, the size distributions follow a Gaussian distribution and are approximately monodispersed (sharply peaked around the mean).

**Figure 5:**
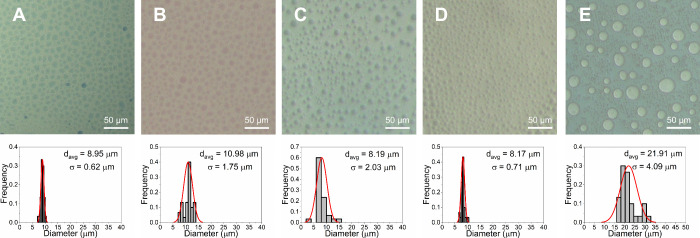
Microscale colloidal phase behavior and size distribution of PTFA-water mixtures at *T*_sep_ = 70 °C and different IL concentrations: (A) 10 wt %, (B) 20 wt %, (C) 30 wt %, (D) 40 wt %, and (E) 50 wt %. The ILR phase (darker color) is dispersed as discontinuous aggregates within a continuous WR phase (lighter color) in panels (A–D), and vice versa in panel (E).

**Figure 6:**
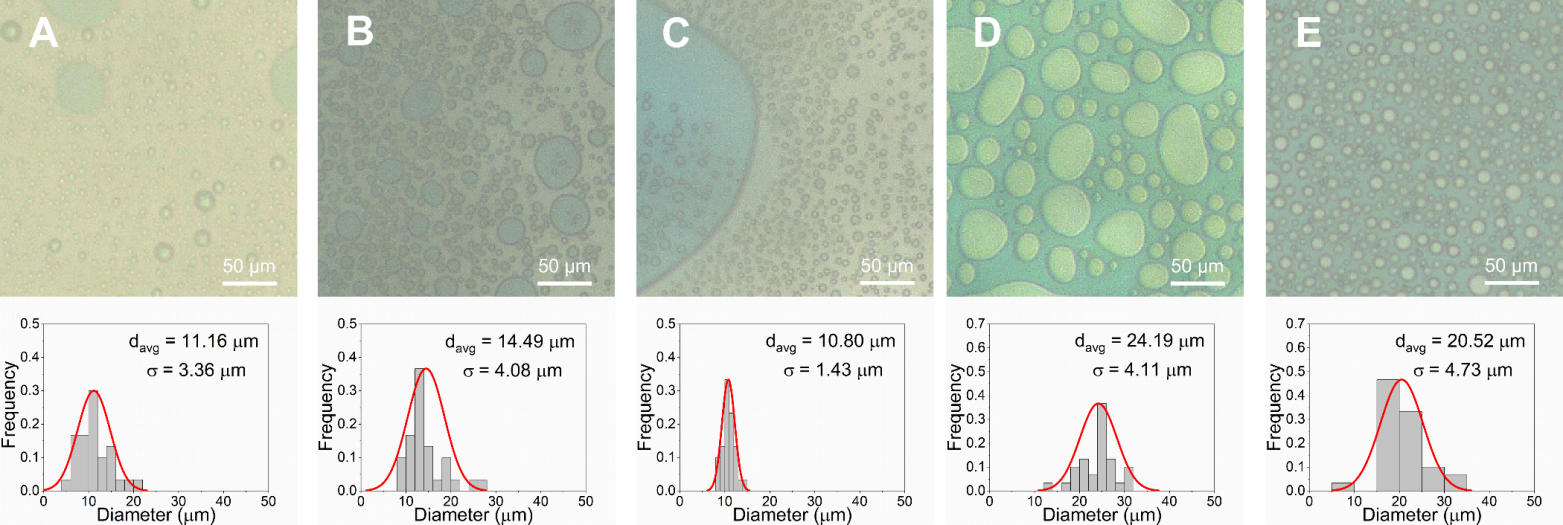
Microscale phase behavior and size distribution of NSal–water mixtures at *T*_sep_ = 70 °C and different IL concentrations: (A) 10 wt %, (B) 20 wt %, (C) 30 wt %, (D) 40 wt %, and (E) 50 wt %. The ILR phase (darker color) is dispersed as discontinuous aggregates within a continuous WR phase (lighter color) in panels (A, B), and vice versa in panel (E). In panel (C), the PMR is less than 2.75, resulting in the formation of discontinuous WR and ILR phases. Size distributions correspond to the phase of the greatest colloidal population, with (C) showing the size distribution of the WR phase, whereas panels (A, B, and E) show the size distribution of the ILR phase.

At a concentration of 10–20 wt %, the four IL-water mixtures have a PMR > 2.75 and hence only the ILR phase forms discontinuous aggregates, as shown in Figures 5A,B and 6A,B for PTFA and NSal, respectively. At these concentrations, NSal has the largest mean size (∼11–14.5 μm) and standard deviation (∼3.4–4 μm) relative to the other ILs. From an application standpoint, this microscale phase behavior is highly desirable coalescence of the discontinuous ILR phase can be accelerated using a centrifuge to rapidly form the macroscopic WR and ILR phases. Interestingly, this behavior is analogous to ternary oil–water–surfactant Winsor III emulsions, which are common in the oil and gas industry.^56–59^

At a concentration of 30–40 wt %, the microscale phase behavior of the four ILs becomes more complex. At 30 wt %, NSal (PMR ≈ 1.5) forms discontinuous aggregates of both the WR and ILR phases (∼11 μm), as shown in Figure 6C. At 40 wt %, however, NSal (PMR ≈ 0.7) transitions toward a discontinuous WR phase (∼24 μm) within a continuous ILR phase, as shown in Figure 6D. For PTFA, on the other hand, despite the fact that PMR is ∼2.5 at 30 wt %, only the ILR phase (∼8 μm) forms discontinuous aggregates, as shown in Figure 5C. At 40 wt %, PTFA (PMR ≈ 1.0) retains this strictly discontinuous ILR phase, as shown in Figure 5D, in contrast to PDMBS that exhibits discontinuous aggregates of either phase (2.75 > PMR > 0.75) and NSal and PSal that form a strictly discontinuous WR phase (PMR < 0.75) as shown in Figures 6D and S13D, respectively.

Increasing the concentration to 50 wt % results in the formation of a strictly discontinuous WR phase in a continuous ILR phase for all four materials (PMR < 0.75), as shown in Figure S14. Further increasing the concentration corresponds to low PMR values below ∼0.2,^28^ which in turn results in the formation of an almost negligible amount of the WR phase at *T*_sep_ = 70 °C.

From an application standpoint, the presence of discontinuous aggregates of either phase complicates the macroscopic coalescence of the WR and ILR phases, since unidirectional centrifugation would accelerate the coalescence of one of the two phases while hindering the coalescence of the other phase. This, in turn, highlights an advantage of using PTFA over the other three ILs, as the phase separation process can be reliably expedited using centrifugation at a much broader IL concentration range relative to NSal, PDMBS, and PSal. It is also worth noting that the phosphonium cation ([P_4444_]^+^) results in smaller-sized colloidal aggregates than its ammonium counterpart ([N_4444_]^+^) when both cations are paired with the same Salicylate-based anion ([Sal]); this comparison of NSal and PSal is shown in Supporting Note 8. However, further investigation of other ILs is necessary to validate if this behavior is universal (i.e., applicable to all phospohonium-based ILs compared to ammonium-based ones for a given anion). With this insight into the size distribution of IL-water mixtures at concentrations between 10 and 50 wt % and *T*_sep_ = 70 °C, we can bridge the gap between the microscopic phase separation and the macroscopic kinetics as discussed in the next section.

### Macroscale Phase Separation Kinetics of LCST ILs

To approximate the macroscale phase separation time and separator size based on the microscale behavior, Stokes’ law is utilized. Depending on the PMR and when *T* ≥ *T*_C_, discontinuous aggregates of a nearly monodispersed mean size of one or both phases will form that either settle at the bottom or float to the top, thereby forming two macroscopically distinct WR and ILR phases as illustrated in Figure 7A. Due to the small size (∼1–10 μm) of the colloidal aggregates and the absence of external velocity gradients or forced convection during phase separation (i.e., in the absence of centrifugation), the settling/rising of such particles is driven solely by the density difference between the WR and ILR phases. Namely, the WR phase aggregates are naturally buoyant in the ILR phase due to their lower density, resulting in the gradual coalescence of the macroscopic WR phase above the ILR phase, whereas the ILR phase aggregates are of a higher density relative to the WR phase and will settle below the WR phase, as illustrated in Figure 7.

**Figure 7:**
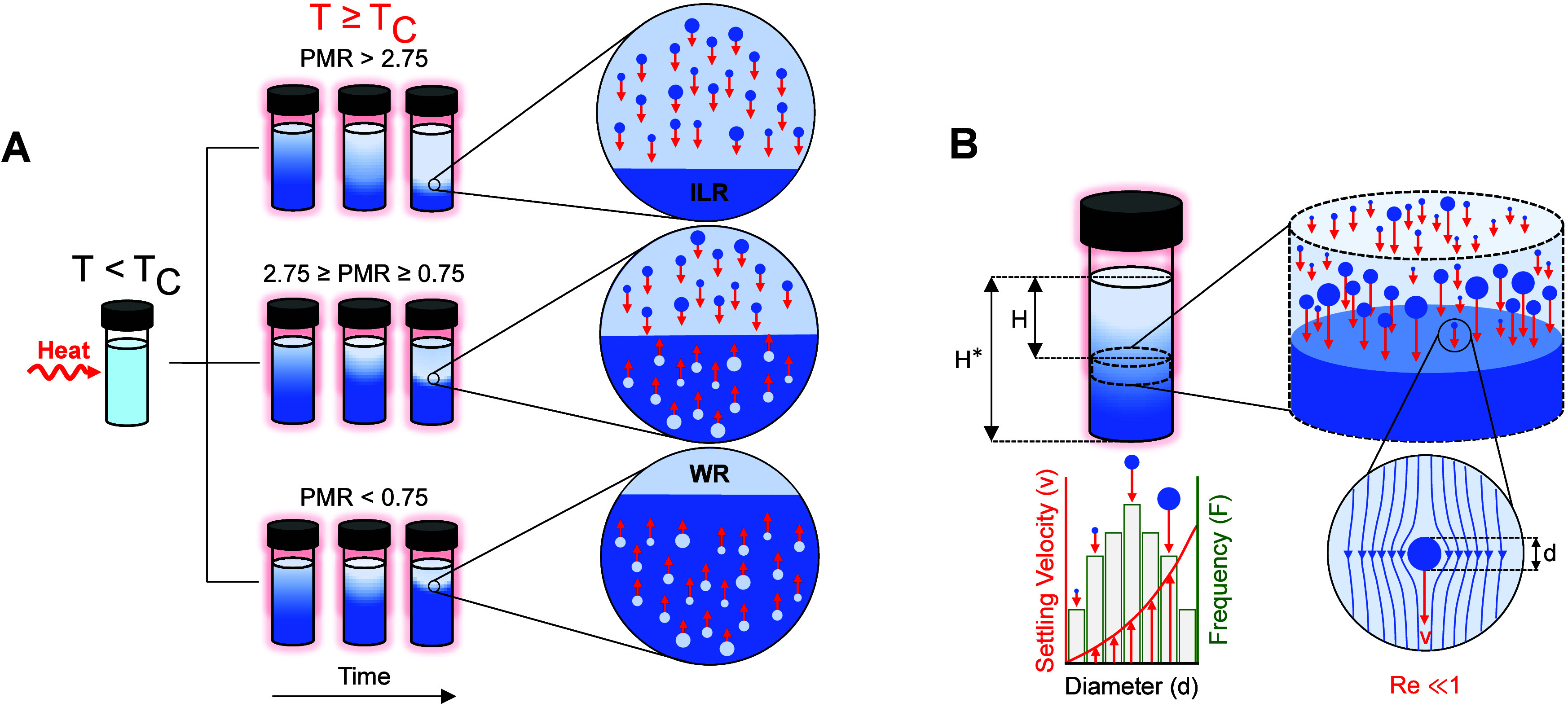
Illustration of the phase separation behavior across length scales. (A) Microscale phase behavior of IL-water mixtures depending on the PMR for a given concentration, upon heating to *T*_C_. (B) Viscous Stokes settling (Re ≪ 1) of discontinuous phasic colloids in a solution. *H* corresponds to the distance between the top of the solution and the phase interface for ILR aggregates settling into the ILR phase (as shown), or the distance between the bottom of the solution and the phase interface for WR aggregates rising toward the WR phase (not shown). H* corresponds to the total solution height.

The Reynolds number (Re) during the phase separation process is ∼10^–6^–10^–7^; since Re ≪ 1,^60^ the flow around the aggregates is viscous with no inertial component, as illustrated in Figure 7B. Since the flow regime is strictly viscous, Stokes’ law can be used to approximate the terminal velocity of a particle settling/rising during the phase separation process.^61^ Whereas Stokes’ law is classically formulated for solid spheres, under sufficiently low Re numbers (Re < 0.5), internal recirculation within a liquid or a gas spherical particle is negligible due to the viscous nature of the flow, resulting in solid particle-like behavior.^62^ It is important to note, however, that even under low Re number conditions, significant deviations from Stokesian behavior can occur if the aggregates are charged, highly irregular in shape, and/or highly polydispersed (one to 2 orders of magnitude).^63–65^ In the absence of such conditions, Stokes’ law can be used to predict the terminal velocity of particles in viscous flow regimes with reasonable accuracy (within10–20%) using the “single particle velocity” prediction approach, i.e., approximating the terminal velocity of multiple particles of different but nearly monodispersed sizes based on the mean particle size.^66–68^

Accordingly, for a particle moving toward the phase interface, the terminal velocity, *v̅*, can be approximated using Stokes’ law shown in eq 1.

$$\overset{-}{v}\approx \frac{{\overset{-}{d}}^{2}\Delta \rho g}{18\mu_{c}}$$ 1

where *d̅*, is the mean colloidal size for a given IL at a given concentration, Δρ is the density difference between the WR and ILR phases, μ_c_ is the dynamic viscosity of the continuous phase around the aggregates, and *g* is the gravitational acceleration. For a given number of settling or rising particles, the time elapsed until all droplets reach the phase interface, *t*_*i*_ Can be expressed following the “single particle velocity” prediction approach as shown in eq 2.^66^

$$t_{i}=\{h_{1}/\overset{-}{v},h_{2}/\overset{-}{v},\cdot \cdot \cdot ,h_{i}/\overset{-}{v},\cdot \cdot \cdot ,h_{n}/\overset{-}{v}\}$$ 2

where *h*_*i*_ is the distance traversed by a given particle to the phase interface. The macroscopic phase separation time (*t*_sep_) that is experimentally observable depends on the maximum of *t*_*i*_, corresponding to the longest distance traversed by a given particle, i.e., *H*, as illustrated in Figure 7B. The macroscopic phase separation, *t*_sep_, can then be calculated theoretically using eq 3.

$$t_{\mathrm{sep}}\approx \max[t_{i}]=H/\overset{-}{v}$$ 3

To determine the experimental value for the macroscopic phase separation time, the spatially averaged transmittance profiles over the WR phase are measured (Figure 3 and Experimental Methods section). Transmittance curves of PTFA, PDMBS, NSal, and PSal are shown in Figures S4 −S7 of Supporting Notes 2–5, respectively. Since the colloidal size distributions of all mixtures at a given concentration are Gaussian and nearly monodispersed (peaking around the mean), this transmittance value corresponds approximately to the settling of the bulk of the colloidal population (greater than ∼1.6 standard deviations below the mean of the distribution, i.e., excluding the bottom ∼10% of the population, as shown in Figures S10–S14). These results are consistent with our previous report on the phase separation kinetics of PTFA and NSal at H* = 2 cm.^28^

Figure 8 shows the experimental phase separation times normalized by the traversed phase height, *H*, along with the theoretically calculated normalized values from eqs 1 and 3. These phase separation times are in close agreement with each other (within 18%), confirming the validity of the Stokesian settling approximation for LCST ILs. The uncertainty bands associated with the theoretical phase separation times are predominantly a consequence of the uncertainty propagation from the experimental uncertainty in the phasic density and viscosity measurements (see Supporting Note 7 for a detailed discussion on experimental uncertainty propagation). To further illustrate this, additional experiments were performed by varying the height of the sample, H*, between 2, 3, and 4 cm, as illustrated in Figure S7. The results in Figure S8 show the proportional dependence of phase separation time on the traversed phase height as is dictated by Stokes’ law in eq 3, yielding close agreement between the theoretical and experimental values (within 19%).

**Figure 8:**
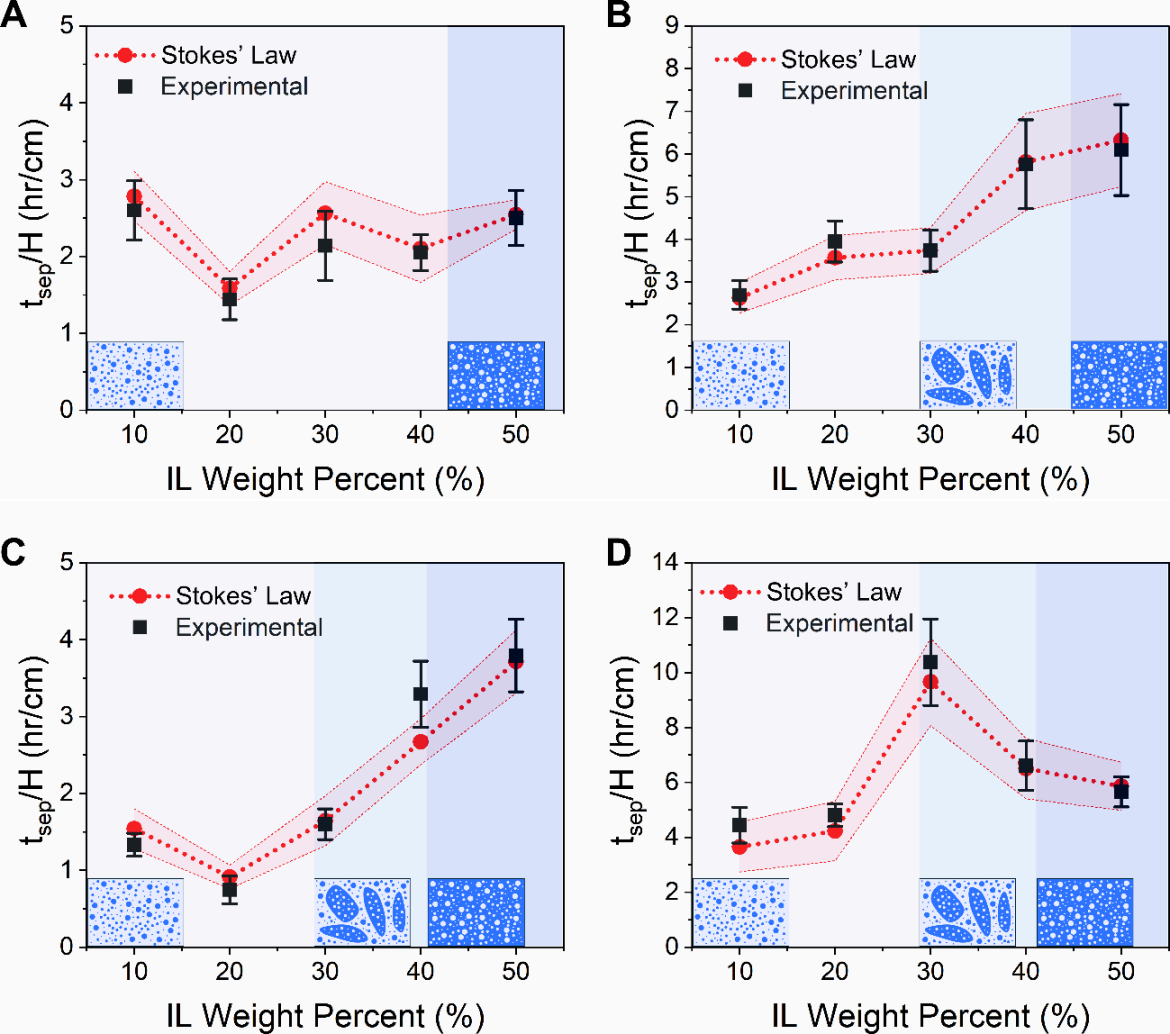
Macroscale phase separation times of the four IL-water mixtures normalized to the traversed phase height, *H*: (A) PTFA, (B) PDMBS, (C) NSal, and (D) PSal. The theoretical phase separation time (red circles) determined from Stokes’ law is based on the average size distribution for all ILs at all concentrations. The error band (shaded region) associated with the theoretical phase separation time corresponds to the experimental uncertainty propagation (Supporting Note 7). The experimental phase separation times (black squares) are determined from position-variable light transmittance measurements. The error bars (black bars) correspond to the standard deviation across three measurements at the onset of reaching 90% of the maximum transmittance. All experiments shown in panels (A–D) were performed at H* = 2 cm and *T*_sep_ = 70 °C. The shaded regions correspond to the specific PMR-dependent regimes illustrated in Figure 4. The shaded regions in panels (A–D) illustrate the microscale behavior of each IL based on the PMR values shown in Figure 4 and their corresponding IL concentrations.^28^

As elucidated by the results shown in this work, each IL exhibits a different kinetic dependence on IL concentration as a consequence of the varying aggregate sizes and concentrations, as shown in Figures S10–S14. This behavior is a consequence of the complex nature of the spinodal decomposition process, which typically has a strong concentration dependence on the concentration of a given LCST (or UCST) species in solution.^69,70^ Herein, we show for the first time that the phase separation time can be approximated as being inversely proportional to the square of the mean colloidal size (i.e., *t*_sep_ ∝ 1/*d̅* ^2^). This highlights the importance of IL phase separation kinetics, which has been neglected thus far in cost analyses of LCST systems.^29,31^

### Understanding the Impact of Phase Separation Kinetics of LCST ILs on Industrial Applications

From an application standpoint, the Stokesian nature of the LCST phase separation process provides a reasonable estimate of the separation time needed for an IL-water mixture at a given concentration. For example, in the case of 30 wt % PTFA, *t*_sep_/*H* is approximately 2 h/cm based on the results shown in Figure 8. For a cylindrical separator of height, H* = 2 m, the traversed phase height, *H*, can be determined from the lever rule (assuming constant cross-sectional area) to be approximately 8.3 days for complete phase separation (unassisted) at *T*_sep_ ***=*** 70 °C.

To further highlight the importance of considering the time scales associated with the phase separation process of a given LCST IL species, we consider a typical static phase separation chamber as reported in recent works in literature in Figure 9A.^9–11,31^ A dilute ILR phase (i.e., *w*_WR_ < *w*_IL_ < *w*_ILR_) flows into a heated chamber at a given *T*_sep_ to initiate the phase separation process. The resulting WR and ILR phases then flow out of the chamber to achieve a useful effect; ILR dilution in the case of FO desalination^11,31^ and dehumidification,^13,15,29,45^ and WR end-use as freshwater in the case of FO desalination^9,31^ or evaporation in the case of LCST evaporative cooling.^15,29^ At a 30 wt % IL concentration and *T*_sep_ = 70 °C, depending on the IL species and separator height, H*, the time needed for phase separation can be on the order of multiple weeks as illustrated in Figure 9B. This reveals that scaling up of technologies utilizing LCST ILs as the working fluid would prove challenging unless the separation process is aided (e.g., using an electrostatic coalescer).

**Figure 9:**
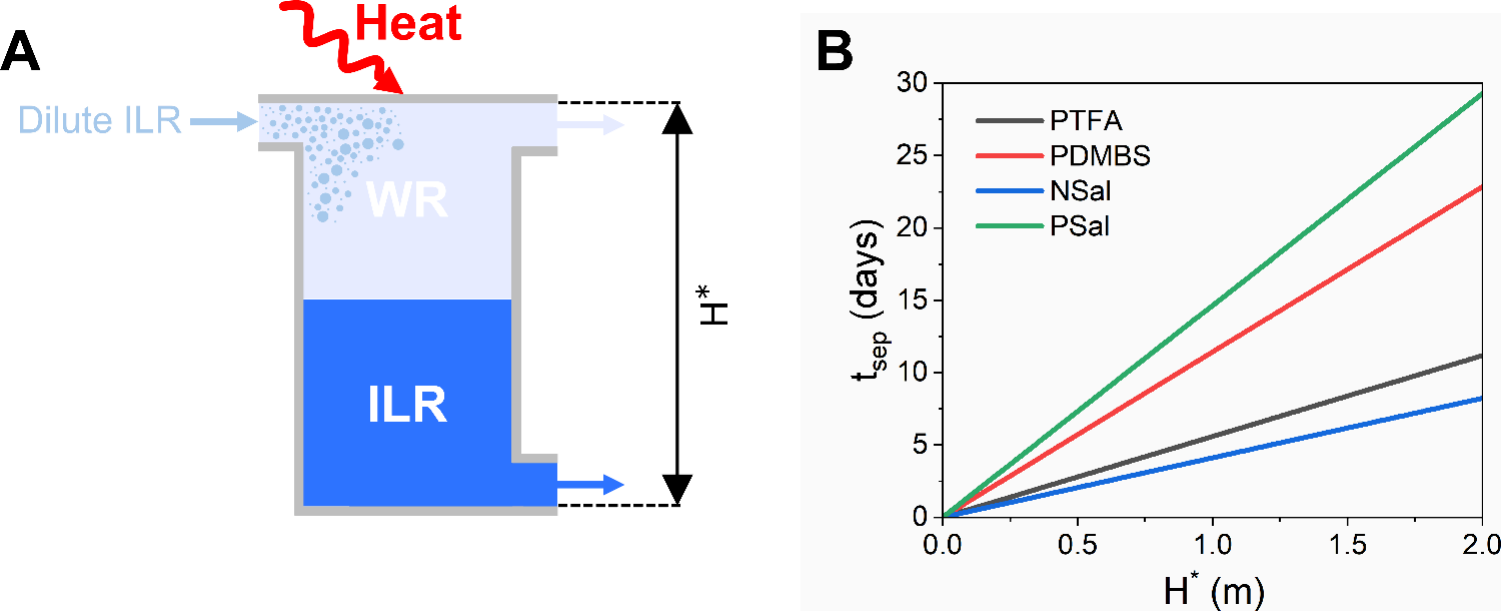
Illustration of phase separation time scales on applications utilizing LCST ILs as a working fluid. (A) Illustration of a typical LCST IL phase separator reported in the literature. (B) Theoretical Stokes’ phase separation times, *t*_sep_, as a function of H* at *T*_sep_ = 70 °C and *w*_IL_ = 30 wt %.

## Conclusions

This work lays the foundation for understanding the macroscale phase separation kinetics of thermally responsive ionic liquids by characterizing their microscale behavior. We show that the microscale behavior of different LCST ILs is governed by their phase mass ratio at a given concentration. Specifically, phase inversion occurs universally for all four ILs reported in this work at PMR < 0.75. The colloidal size distributions are then used to approximate the macroscale phase separation time using Stokes’ law. This, in turn, enables appropriate design and sizing of liquid–liquid separators for different energy-water applications involving LCST ILs. This work also paves the way for theoretical spinodal decomposition modeling based on the aggregate size distributions of PTFA, PDMBS, NSal, and PSal at different concentrations, which addresses a gap in the literature. Future work should focus on modeling the spinodal decomposition behavior of these LCST ILs using phase-field equations to better understand the governing parameters that drive the formation of aggregates of different size distributions as a function of chemistry and concentration. Particular focus should be set on accelerating the separation time through centrifugation or convective flow, to mitigate the slow kinetics inherent with Stokesian flow.

## Supplementary Material



## Data Availability

The data supporting this article have been included as part of the Supporting Information.

## Notes

Viscosity and density measurements (Supporting Note 1), PTFA phase separation kinetics (Supporting Note 2), PDMBS phase separation kinetics (Supporting Note 3), NSal phase separation kinetics (Supporting Note 4), PSal phase separation kinetics (Supporting Note 5), height-dependent separation kinetics (Supporting Note 6), uncertainty propagation analysis (Supporting Note 7), and microscopic images of the microscale colloidal phase behavior (Supporting Note 8) (PDF)
