# Supplementary material for: Elucidating the Microscale Behavior and Phase Separation Kinetics of Thermally Responsive Ionic Liquid–Water Mixtures
Source: ACS Appl Mater Interfaces. 2026 Feb 12;18(7):12177–87. doi: 10.1021/acsami.5c24522 (PMC12954671; doi:10.1021/acsami.5c24522)
Supplement: Supplementary file 1 [file am5c24522_si_001.pdf]

Supporting Information

Elucidating the Microscale Behavior and Phase Separation Kinetics of Thermally Responsive Ionic Liquid-Water Mixtures

Ahmed Mahfouz[a], Jordan D. Kocher[a], Andrew Z. Haddad[b], and Akanksha K. Menon*[a]

a. George W. Woodruff School of Mechanical Engineering, Georgia Institute of Technology, Atlanta, GA 30332, USA

b. Energy Storage and Distributed Resources Division, Lawrence Berkeley National Laboratory, Berkeley, CA 94720, USA

* Corresponding author. Email: akanksha.menon@me.gatech.edu

Supporting Note 1: Viscosity and Density Measurements

The viscosity and density measurements performed for the WR and WS phases of PTFA, PDMBS, NSal, and PSal are reported. A 10 g 40 wt.% aqueous solution of each of the IL species is prepared and phase separated at T_{sep} = 70 °C for 24 hours. The two phases are pipetted into two separate vials and are allowed to cool down to 50 °C to meet the temperature limit of the Densito density meter as illustrated in Fig. S1A. Given that the IL solutions are incompressible, measuring the density at 50 °C instead of 70 °C impacts the density measurements negligibly (<2%) [1]. The density of each phase is measured three times to calculate the phase density difference, $\Delta\rho$, between either phase. Fig. S2 shows the measured average phase density difference of the four IL species studied in this work at T_{sep} = 70 °C.

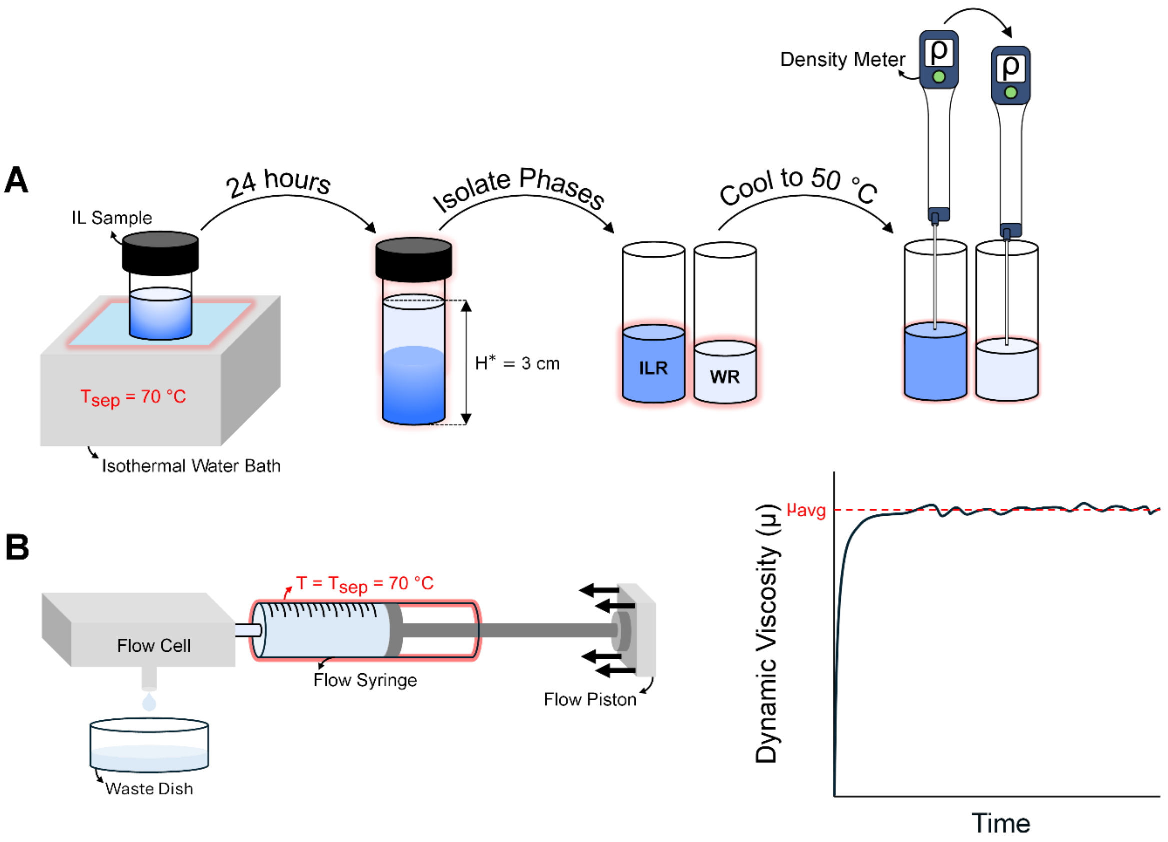

Fig. S1. Experimental viscosity and phase density measurement procedure. **A** Phase density. **B** RheoSense m-VROC viscosity measurement procedure. To perform the density measurements as illustrated in **A**, the ILR and WR phases are phase separated at 70 °C, cooled down to the maximum measurement temperature of the Mettler Toledo Densito density meter of 50 °C. Each density measurement is repeated three times.

The dynamic viscosity of the WR and ILR phases of either phase of the four IL species was characterized using the RheoSense m-VROC viscometer. Given that the ILs exhibit a Newtonian viscosity-shear rate trend, the dynamic viscosity is independent of the shear rate [2, 3]. As such, the viscosity of either phase is measured at a constant shear rate of 1000 s^{-1} for 2 minutes at $T = T_{sep}$ = 70 °C as illustrated in Fig. S1B. The dynamic viscosity measurement results are shown in Fig. S3.

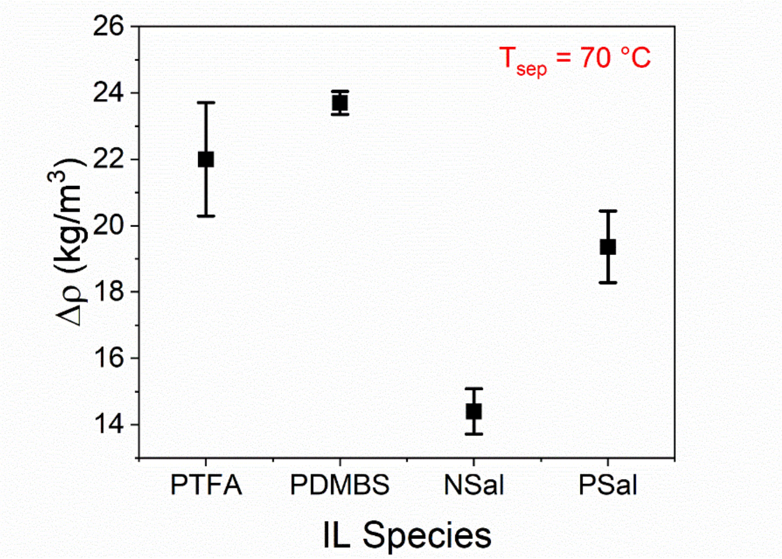

Fig. S2. Phase density difference, $\boldsymbol{\Delta\rho}$, at 50 °C between the WR and the ILR phases at $\boldsymbol{T_{sep}}$ = 70 °C. Error bars correspond to the standard deviation between three measurements.

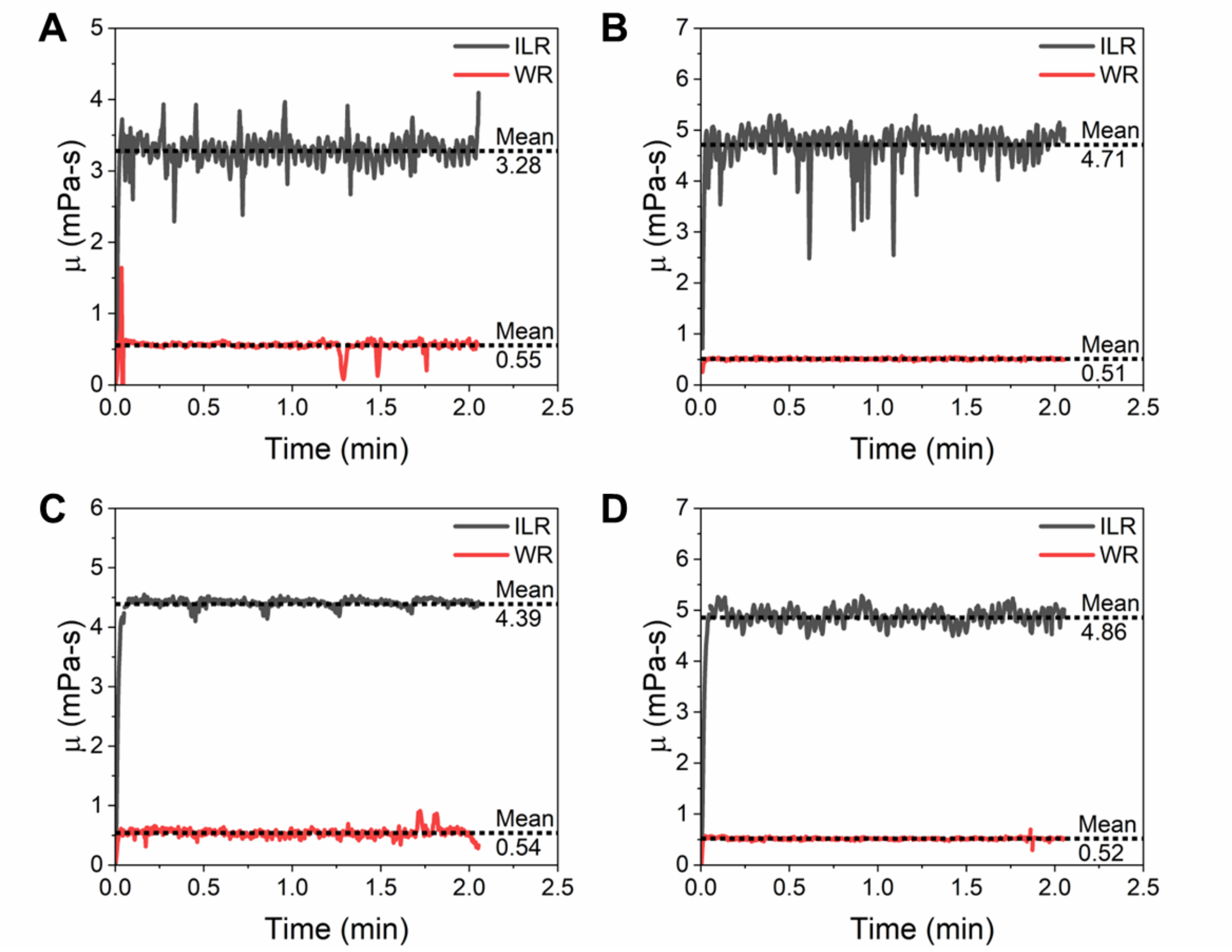

Fig. S3. Viscosity measurements at 70 °C of the WR and ILR phases of all four ILs at $\boldsymbol{T_{sep}}$ = 70 °C. **A** PTFA. **B** PDMBS. **C** NSal. **D** PSal.

Supporting Note 2: PTFA Phase Separation Kinetics

The time-dependent transmittance profiles are shown, which are averaged over the WR phase, with three independent trials for PTFA at different concentrations and H^* values. Fig. S4A to D show the transmittance profiles for 10, 20, 40, and 50 wt.% respectively at $H^* = 2$ cm whereas Fig. S4E to F show the transmittance profiles for PTFA at 30 wt.% concentration $H^* = 3$ cm and 4 cm, respectively. Due to the stochastic nature of the phase separation process, macroscopic droplet movement that interferes with the light source in one of the three trials results in large and abrupt drops in transmittance, as in the case of Fig. S4B and D, due to the diffraction of some of the light signal away from the light detector [4].

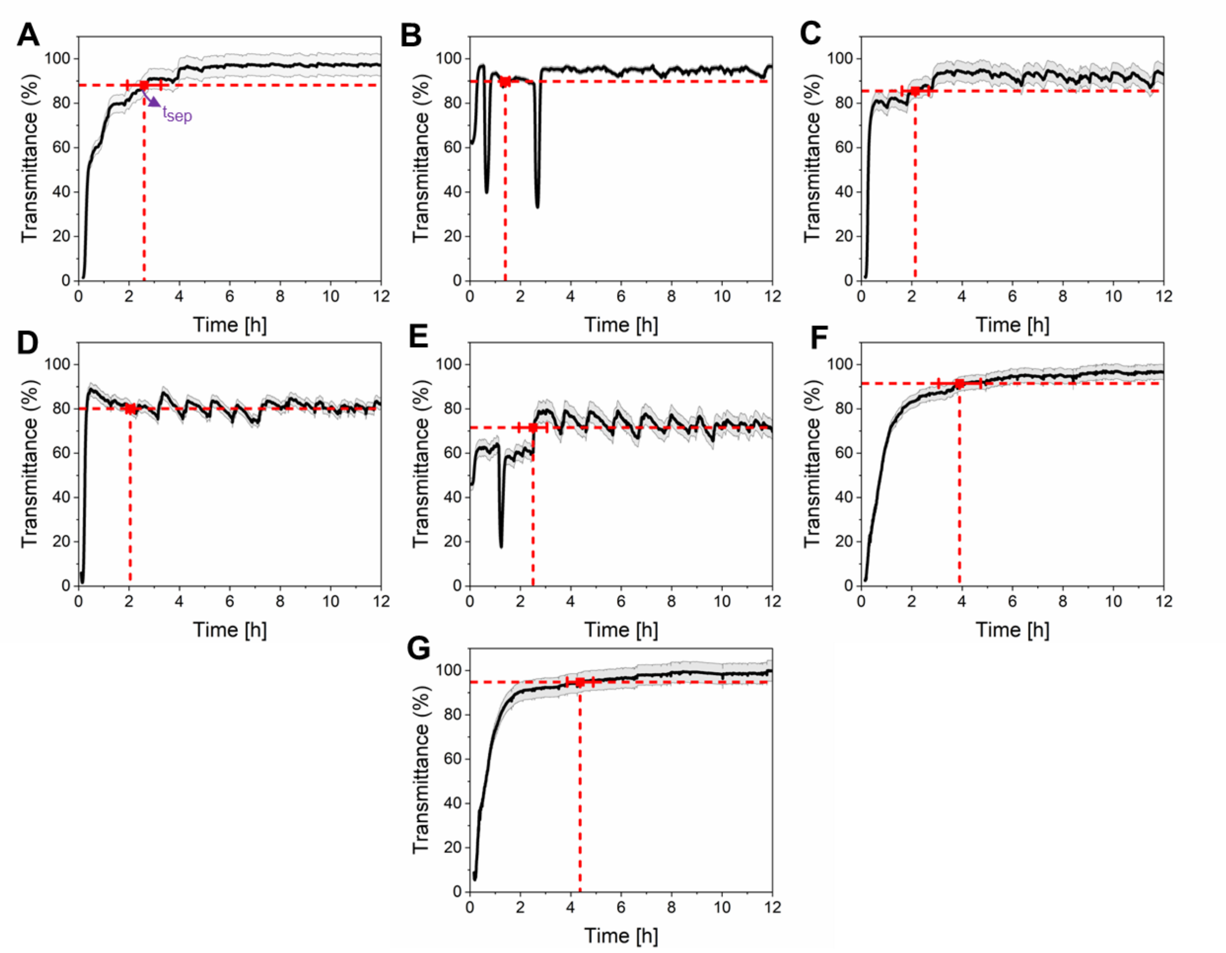

Fig. S4. Time-dependent percent transmittance curves at 470 nm, spatially averaged over the WR phase and over three measurements for PTFA at different concentrations at $\boldsymbol{T_{sep}} = 70$ °C. **A** 10 wt.%, **B** 20 wt.%, **C** 30 wt.%, **D** 40 wt.%, **E** 50 wt.%, **F** and **G** 30 wt.%. For **A** to **E**, $\boldsymbol{H^*} = 2$ cm, while for **F** and **G**, $\boldsymbol{H^*}$ is 3 cm and 4 cm, respectively. The error band (light-shaded region) represents the maximum standard deviation between three trials. The error bar of the experimental phase separation time, $\boldsymbol{t_{sep}}$, represents the standard deviation between three measurements when the transmittance reaches 90% of the maximum value.

Supporting Note 3: PDMBS Phase Separation Kinetics

The time-dependent transmittance profiles are shown, which are averaged over the WR phase, with three independent trials for PDMBS at different concentrations and H^* values. Fig. S4A to D show the transmittance profiles for 10, 20, 40, and 50 wt.% respectively at $H^* = 2$ cm whereas Fig. S4E to F show the transmittance profiles for PDMBS at 30 wt.% concentration $H^* = 3$ cm and 4 cm, respectively.

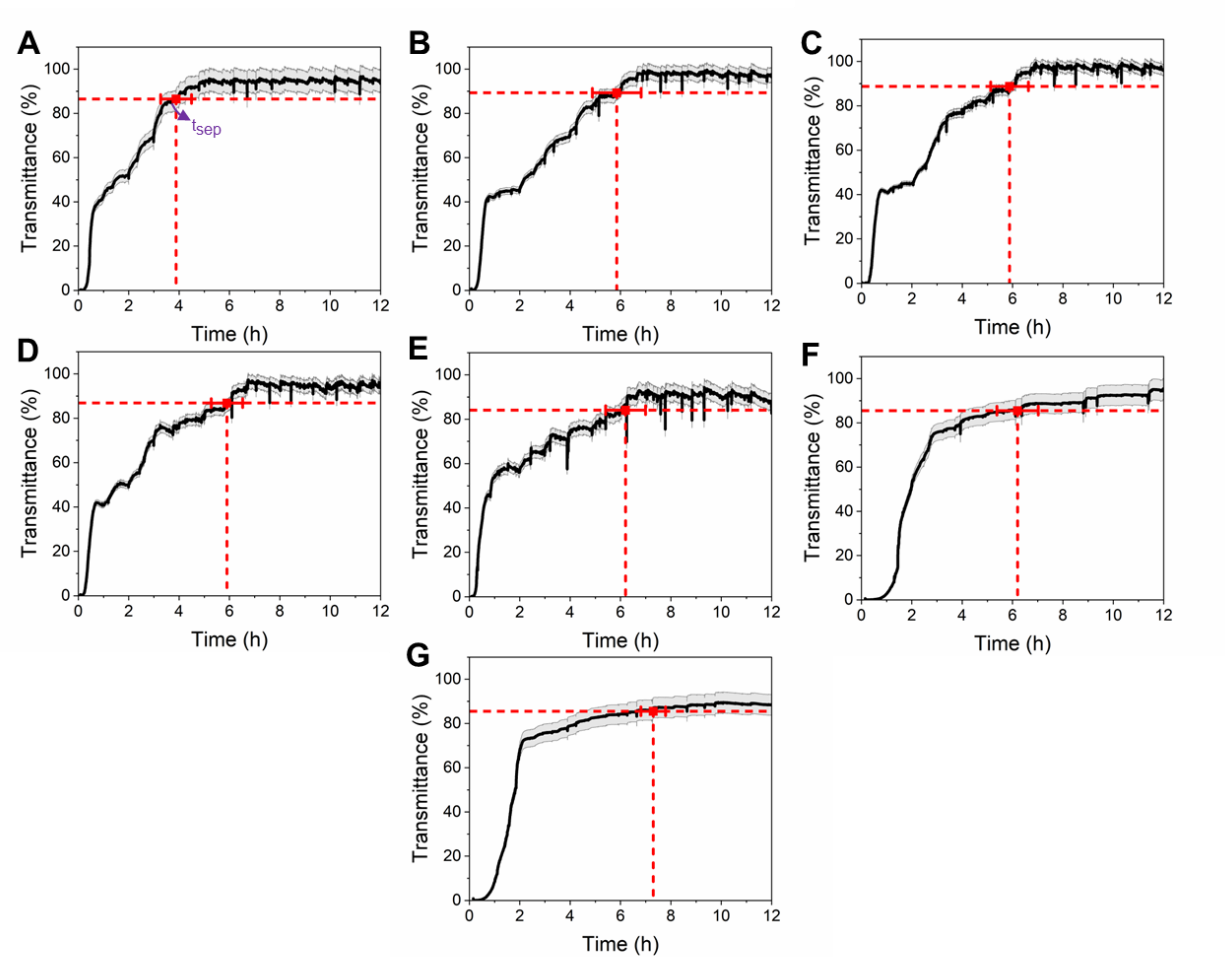

Fig. S5. Time-dependent percent transmittance curves at 470 nm, spatially averaged over the WR phase and over three measurements for PDMBS at different concentrations at $\boldsymbol{T_{sep}} = 70$ °C. **A** 10 wt.%. **B** 20 wt.%. **C** 30 wt.%. **D** 40 wt.%. **E** 50 wt.% **F** and **G** 30 wt.%. For **A** to **E**, $\boldsymbol{H^*} = 2$ cm, while for **F** and **G**, $\boldsymbol{H^*}$ is 3 cm and 4 cm, respectively. The error band (light-shaded region) represents the maximum standard deviation between three trials. The error bar of the experimental phase separation time, $\boldsymbol{t_{sep}}$, represents the standard deviation between three measurements when the transmittance reaches 90% of the maximum transmittance.

Supporting Note 4: NSal Phase Separation Kinetics

The time-dependent transmittance profiles are shown, which are averaged over the WR phase, with three independent trials for NSal at different concentrations and H^* values. Fig. S4A to D show the transmittance profiles for 10, 20, 40, and 50 wt.% respectively at $H^* = 2$ cm whereas Fig. S4E to F show the transmittance profiles for NSal at 30 wt.% concentration $H^* = 3$ cm and 4 cm, respectively.

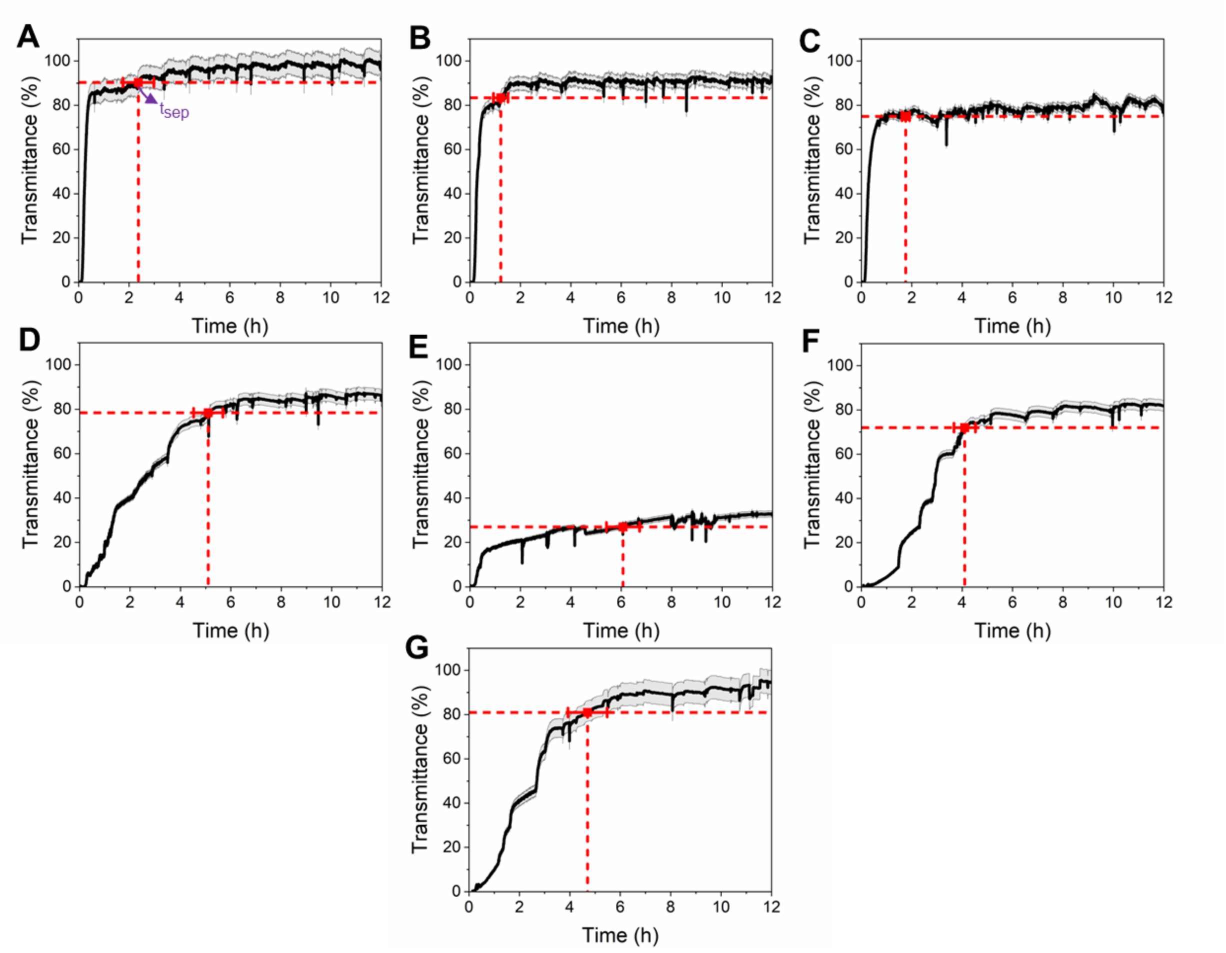

Fig. S6. Time-dependent percent transmittance curves at 470 nm, spatially averaged over the WR phase and over three measurements for NSal at different concentrations at $\boldsymbol{T_{sep}} = 70$ °C. **A** 10 wt.%. **B** 20 wt.%. **C** 30 wt.%. **D** 40 wt.%. **E** 50 wt.% **F** and **G** 30 wt.%. For **A** to **E**, $\boldsymbol{H^*} = 2$ cm, while for **F** and **G**, $\boldsymbol{H^*}$ is 3 cm and 4 cm, respectively. The error band (light-shaded region) represents the maximum standard deviation between three trials. The error bar of the experimental phase separation time, $\boldsymbol{t_{sep}}$, represents the standard deviation between three measurements when the transmittance reaches 90% of the maximum transmittance.

Supporting Note 5: PSal Phase Separation Kinetics

The time-dependent transmittance profiles are shown, which are averaged over the WR phase, with three independent trials for PSal at different concentrations and H^* values. Fig. S4A to D show the transmittance profiles for 10, 20, 40, and 50 wt.%, respectively at $H^* = 2$ cm whereas Fig. S4E to F show the transmittance profiles for PSal at 30 wt.% concentration $H^* = 3$ cm and 4 cm, respectively.

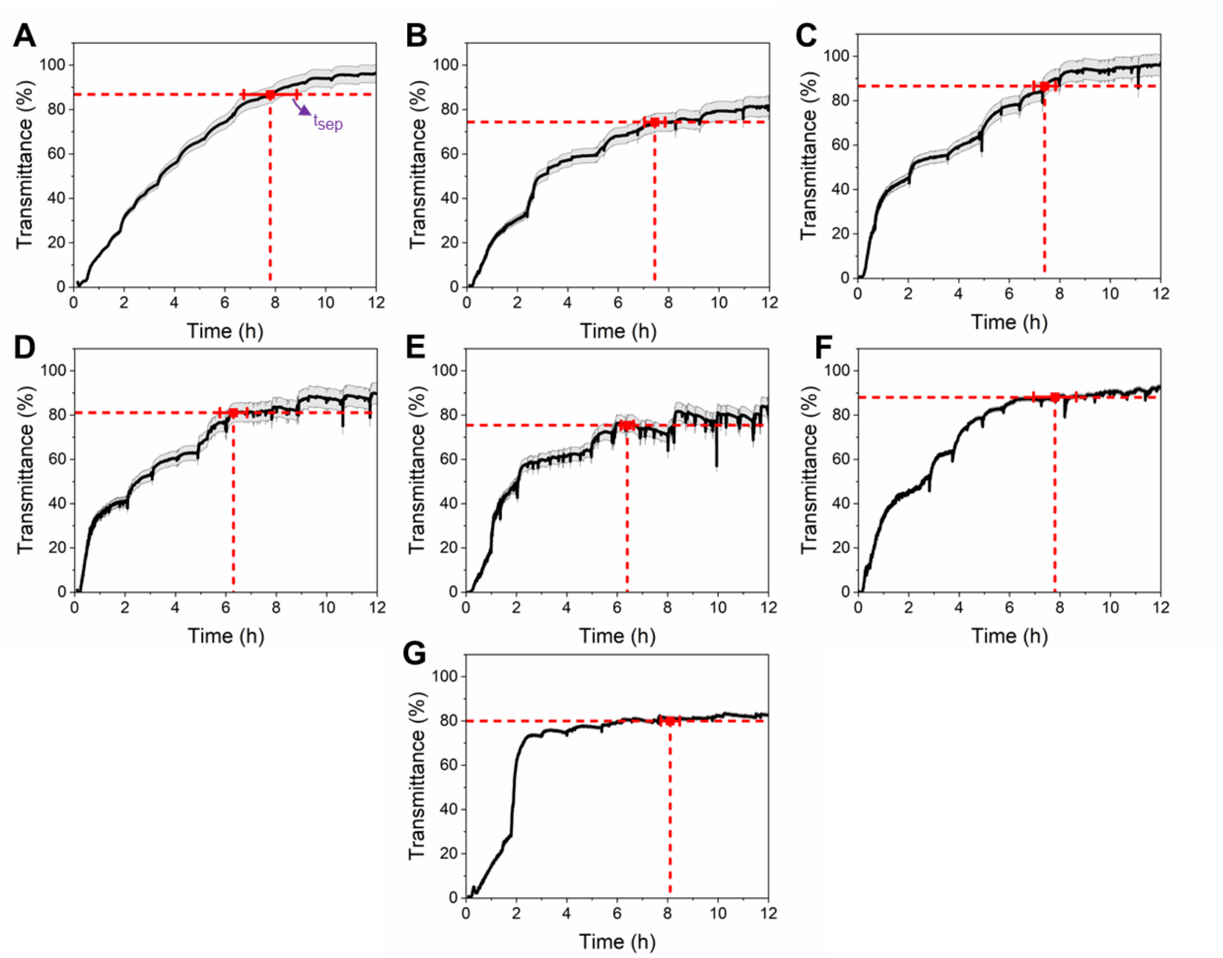

Fig. S7. Time-dependent percent transmittance curves at 470 nm, spatially averaged over the WR phase and over three measurements for PSal at different concentrations at $\boldsymbol{T_{sep}} = 70$ °C. **A** 10 wt.%. **B** 20 wt.%. **C** 30 wt.%. **D** 40 wt.%. **E** 50 wt.% **F** and **G** 30 wt.%. For **A** to **E**, $\boldsymbol{H^*} = 2$ cm, while for **F** and **G**, $\boldsymbol{H^*}$ is 3 cm and 4 cm, respectively. The error band (light-shaded region) represents the maximum standard deviation between three trials. The error bar of the experimental phase separation time, $\boldsymbol{t_{sep}}$, represents the standard deviation between three measurements when the transmittance reaches 90% of the maximum transmittance.

Supporting Note 6: Height-Dependent Separation Kinetics

To demonstrate the dependence of the phase separation time on the phase height, H, as predicted according to Stokes' settling law, a series of transmittance experiments at total heights, H^*, of 2, 3, and 4 cm, are performed for each of the four ILs at 30 wt.% concentration and $T_{sep} = 70$ °C as illustrated in Fig. S8. The transmittance profiles for $H^* = 2$ cm for all four ILs are shown in Figure 12 of the main text, while the $H^* = 3$ cm and $H^* = 4$ cm cases for PTFA, PDMBS, NSal, and PSal are shown in Fig. S4E and F, Fig. S5 E and F, Fig. S6, and Fig. S7E and F, respectively. The theoretical Stokes and experimental settling times are shown in Fig. S9 for all H^* and IL species. The theoretical and experimental settling times exhibit close quantitative and qualitative agreement. The theoretical settling time is modeled based on the average droplet size using Eq. (3) of the main manuscript.

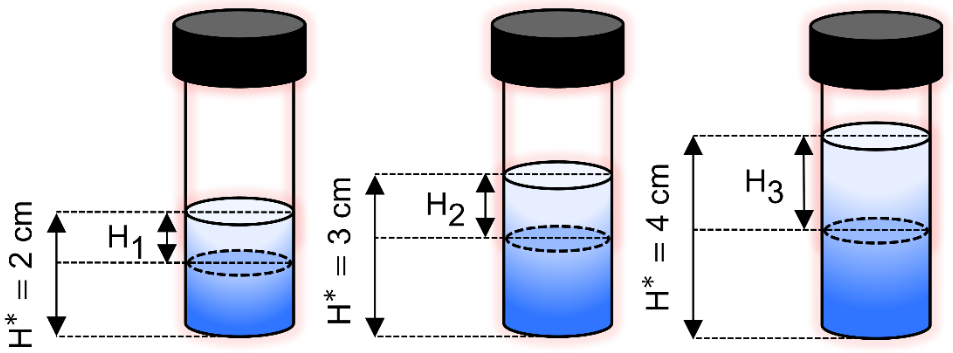

Fig. S8. Illustration of the height variation experiment to assess the dependency of phase height, $\boldsymbol{H}$, on the phase separation kinetics at $\boldsymbol{T_{sep}} = 70$ °C. The total height, $\boldsymbol{H^*}$, is varied between 2, 3, and 4 cm, such that $\boldsymbol{H_3} > \boldsymbol{H_2} > \boldsymbol{H_1}$.

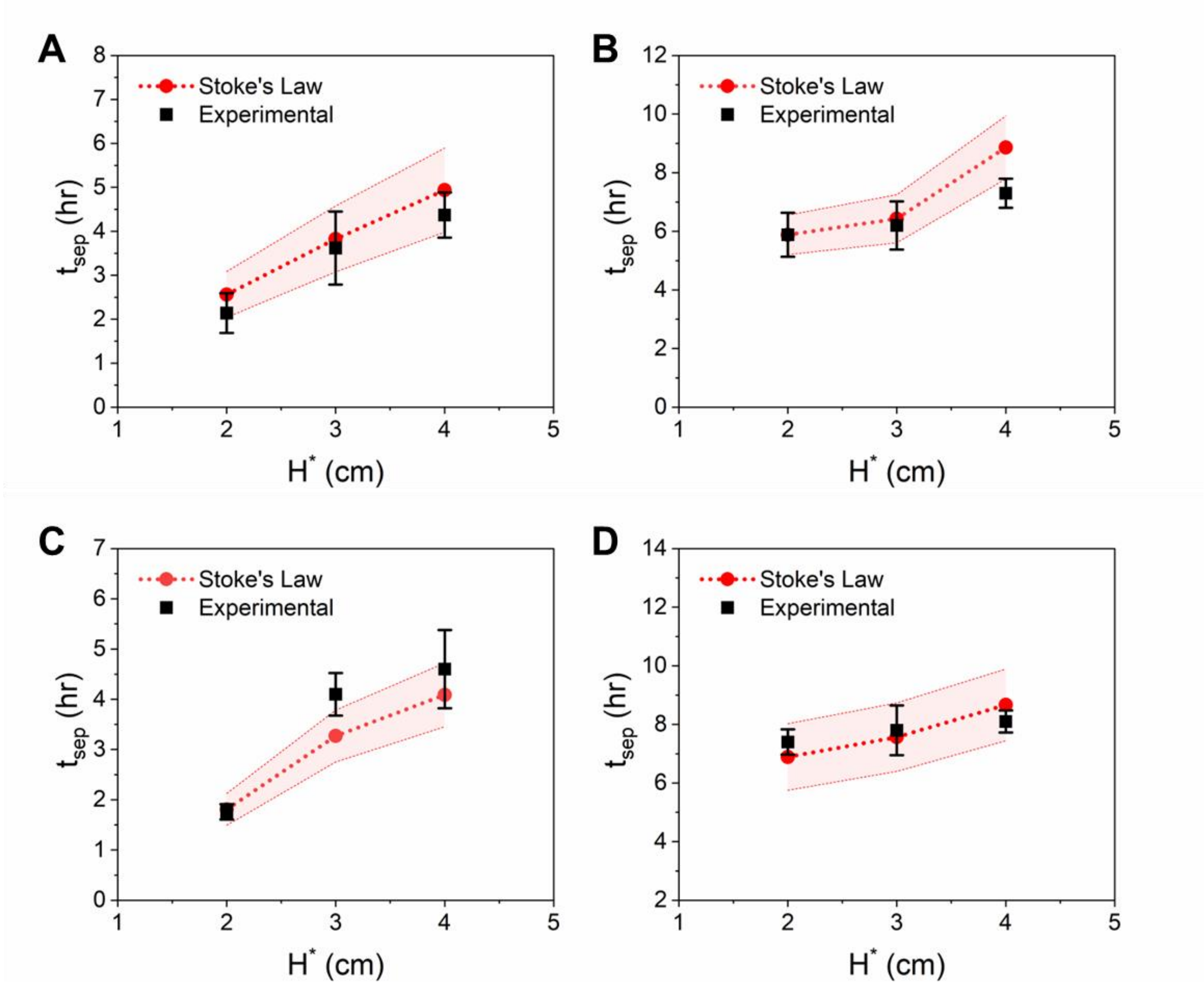

Fig. S9. Stoke's law model validation based on the phase separation times of the four ILs as a function of solution height, $\boldsymbol{H}^*$ at $\boldsymbol{T_{sep}} = 70$ °C. **A** PTFA. **B** PDMBS. **C** NSal. **D** PSal. The IL concentration is 30 wt.% for all four ILs. The theoretical phase separation time determined from Stokes' law is based on the average colloidal size distribution of all ILs at this concentration. The error band associated with the theoretical phase separation time represents the experimental uncertainty propagation (See Supplementary Note 7 for a detailed uncertainty propagation analysis). The error bar of the experimental phase separation time represents the standard deviation between the three trials on the onset of reaching 90% of the maximum transmittance.

Supporting Note 7: Uncertainty Propagation Analysis

The uncertainty propagation is quantified for the theoretical Stokes' law settling time based on the experimental uncertainty in the viscosity, μ_c, density difference between the WR and ILR phases, $\Delta\rho$, the mean colloidal size, $\bar{d}$, traversed phase height, H. The mean Stokes' settling velocity, $\bar{v}$, settling/separation time, t_{sep}, and the separation time normalized by the traversed phase height, τ, are given in Eq. (S1) to (S3), respectively.

$$\bar{v} = \frac{\bar{d}^2 \Delta\rho g}{18\mu_c} \tag{S1}$$

$$t_{sep} = H/\bar{v} \tag{S2}$$

$$\tau = t_{sep}/H \tag{S3}$$

The uncertainty propagation to the theoretically determined values of $\bar{v}$, t_{sep}, and τ, from the experimental measurables, H, μ, $\Delta\rho$, and $\bar{d}$, are given by $\sigma_{\bar{v}}$, $\sigma_{t_{sep}}$, and σ_τ in Eq. (S4) to (S6), respectively. The experimental uncertainty in H^*, μ, $\Delta\rho$, and $\bar{d}$ are given by σ_{H^*}, σ_μ, $\sigma_{\Delta\rho}$, and $\sigma_{\bar{d}}$, respectively.

$$\sigma_{\bar{v}} = \sqrt{\sigma_{\bar{d}}^2 \left(\frac{\partial \bar{v}}{\partial \bar{d}}\right)^2 + \sigma_{\mu_c}^2 \left(\frac{\partial \bar{v}}{\partial \mu_c}\right)^2 + \sigma_{\Delta\rho}^2 \left(\frac{\partial \bar{v}}{\partial \Delta\rho}\right)^2} \tag{S4}$$

$$\sigma_{t_{sep}} = \sqrt{\sigma_H^2 \left(\frac{\partial t_{sep}}{\partial H}\right)^2 + \sigma_{\bar{v}}^2 \left(\frac{\partial t_{sep}}{\partial \bar{v}}\right)^2} \tag{S5}$$

$$\sigma_\tau = \sqrt{\sigma_{t_{sep}}^2 \left(\frac{\partial \tau}{\partial t_{sep}}\right)^2 + \sigma_H^2 \left(\frac{\partial \tau}{\partial H}\right)^2} \tag{S6}$$

Substituting for τ as in Eq. (S2) in Eq. (S6) and taking partial derivatives yields Eq. (S7).

$$\sigma_\tau = \sqrt{\sigma_{t_{sep}}^2 \left(\frac{1}{H}\right)^2 + \sigma_{H^*}^2 \left(\frac{t_{sep}}{H^2}\right)^2} \tag{S7}$$

Substituting for t_{sep} as in Eq. (S2) in Eq. (S5), $\sigma_{t_{sep}}$ as in Eq. (S5) in Eq. (S7), and taking partial derivatives yields Eq. (S8).

$$\sigma_\tau = \sqrt{\left[\sigma_{H^*}^2\left(\frac{1}{\bar{v}}\right)^2 + \sigma_{\bar{v}}^2\left(\frac{H}{\bar{v}^2}\right)^2\right]\left(\frac{1}{H}\right)^2 + \sigma_{H^*}^2\left(\frac{t_{sep}}{H^2}\right)^2} \tag{S8}$$

Substituting for σ_v as in Eq. (S4) in Eq. (S8), yields Eq. (S9).

$$\sigma_\tau = \sqrt{\left[\sigma_H^2\left(\frac{1}{\bar{v}}\right)^2 + \left[\sigma_{\bar{d}}^2\left(\frac{\partial\bar{v}}{\partial\bar{d}}\right)^2 + \sigma_{\mu_c}^2\left(\frac{\partial\bar{v}}{\partial\mu_c}\right)^2 + \sigma_{\Delta\rho}^2\left(\frac{\partial\bar{v}}{\partial\Delta\rho}\right)^2\right]\left(\frac{H}{\bar{v}^2}\right)^2\right]\left(\frac{1}{H}\right)^2 + \sigma_H^2\left(\frac{t_{sep}}{H^2}\right)^2} \tag{S9}$$

Substituting for t_{sep} as in Eq. (S2), for $\bar{v}$ as in Eq. (S1), and taking the partial derivatives, yields Eq. (S10) to (S13).

$$\sigma_\tau = \sqrt{A + B + C} \tag{S10}$$

$$\mathrm{A} = \left(\frac{\sigma_H 18\mu_c}{\bar{d}^2 \Delta\rho g H}\right)^2 \tag{11}$$

$$\mathrm{B} = \left[\sigma_{\bar{d}}^2\left(\frac{\bar{d}\Delta\rho}{9}\right)^2 + \sigma_{\mu_c}^2\left(\frac{\bar{d}^2\Delta\rho}{18\mu_c}\right)^2 + \sigma_{\Delta\rho}^2\left(\frac{\bar{d}^2}{18}\right)^2\right]\left(\frac{324\mu_c}{\left(\bar{d}^2\Delta\rho\right)^2 g}\right)^2 \tag{S12}$$

$$C = \left(\frac{18\mu_c\sigma_H}{H^*\bar{d}^2\Delta\rho g}\right)^2 \tag{S13}$$

Eq. (S10) to (S13) explicitly express the uncertainty propagation to the theoretically determined value of τ from the experimental measurables and their respective uncertainties. These equations are used to calculate the theoretical uncertainty bands shown in Figure 13 of the main text. The digital resolution of the microscopic images shown in this work is 0.3 μm/pixel, as such, the maximum uncertainty in determining $\bar{d}$ experimentally is $\sigma_{\bar{d}} \approx$ 0.6 μm. The maximum experimental uncertainty in determining the phase height, as illustrated in Figure 2B of the main text, is equivalent to the phase boundary depth, which is approximately 1 mm across all IL samples in this study, hence, $\sigma_{H^*} \approx$ 1 mm. The experimental uncertainties of the μ and $\Delta\rho$ measurements shown in Fig. S2 and Fig. S3 of Supplementary Note 1 are tabulated in

Table S1.

Table S1. Measured $\boldsymbol{\mu}$ and $\boldsymbol{\Delta\rho}$ values reported in Fig. S2 and Fig. S3 at $\boldsymbol{T_{sep}}$ = 70 °C and their associated uncertainties. All of viscosity and density measurements are performed at 70 °C and 50 °C, respectively.

	**PTFA**	**PDMBS**	**NSal**	**PSal**
Phase Density Difference (kg/m^3)				
$\Delta\rho$	22.00	23.70	14.40	19.36
$\sigma_{\Delta\rho}$	1.70	0.35	0.68	1.10
Phase Viscosity (Pa-s)				
μ_{WR}	$5.42\cdot10^{-4}$	$5.16\cdot10^{-4}$	$5.50\cdot10^{-4}$	$5.16\cdot10^{-4}$
$\sigma_{\mu_{WR}}$	$9.63\cdot10^{-5}$	$2.95\cdot10^{-6}$	$7.53\cdot10^{-5}$	$1.11\cdot10^{-4}$
μ_{WR}	$3.30\cdot10^{-3}$	$4.89\cdot10^{-3}$	$4.42\cdot10^{-3}$	$4.89\cdot10^{-3}$
$\sigma_{\mu_{WR}}$	$2.55\cdot10^{-4}$	$4.25\cdot10^{-4}$	$2.59\cdot10^{-4}$	$3.39\cdot10^{-4}$

To calculate the theoretical uncertainty bands of the theoretical height-dependent separation time, $\sigma_{t_{sep}}$, shown in Fig. S8, in terms of the experimental measurables and their uncertainties, we substitute for t_{sep} as in Eq. (S2) in Eq. (S5) and take the partial derivatives, yielding Eq. (S13).

$$\sigma_{t_{sep}} = \sqrt{\sigma_{H^*}^2\left(\frac{1}{\bar{v}}\right)^2 + \sigma_{\bar{v}}^2\left(\frac{H}{\bar{v}^2}\right)^2} \tag{S13}$$

Substituting for $\bar{v}$ as in Eq. (S1) and for $\sigma_{\bar{v}}$ as in Eq. (S4) in Eq. (S13) and taking the partial derivatives, yields Eq. (S14), expressing the uncertainty in the theoretical separation, $\sigma_{t_{sep}}$, in terms of the experimental measurables H, μ, $\Delta\rho$, and $\bar{d}$, and their respective uncertainties, σ_{H^*}, σ_{μ}, $\sigma_{\Delta\rho}$, and $\sigma_{\bar{d}}$, respectively.

$$\sigma_{t_{sep}} = \sqrt{\sigma_H^2\left(\frac{18\mu_c}{\bar{d}^2\Delta\rho g}\right)^2 + \left[\sigma_{\bar{d}}^2\frac{\bar{d}\Delta\rho g}{9\mu_c^2} + \sigma_{\mu_c}^2\frac{\bar{d}^2\Delta\rho g}{18\mu_c^2} + \sigma_{\Delta\rho}^2\frac{\bar{d}^2 g}{18\mu_c}\right]\left(\frac{324\mu_c^2 H}{\left(\bar{d}^2\Delta\rho g\right)^2}\right)^2} \tag{S14}$$

Supporting Note 8: Microscopic Images of the Microscale Colloidal Phase Behavior

Microscopic images of the colloidal biphasic behavior of PTFA, PDMBS, NSal, and PSal at concentrations between 10 wt.% to 50 wt.% are shown in Fig. S10 to Fig. S14, as discussed in the text of the main manuscript. Table S2 summarizes the colloidal mean sizes and standard deviations shown in Fig. S10 to Fig. S14.

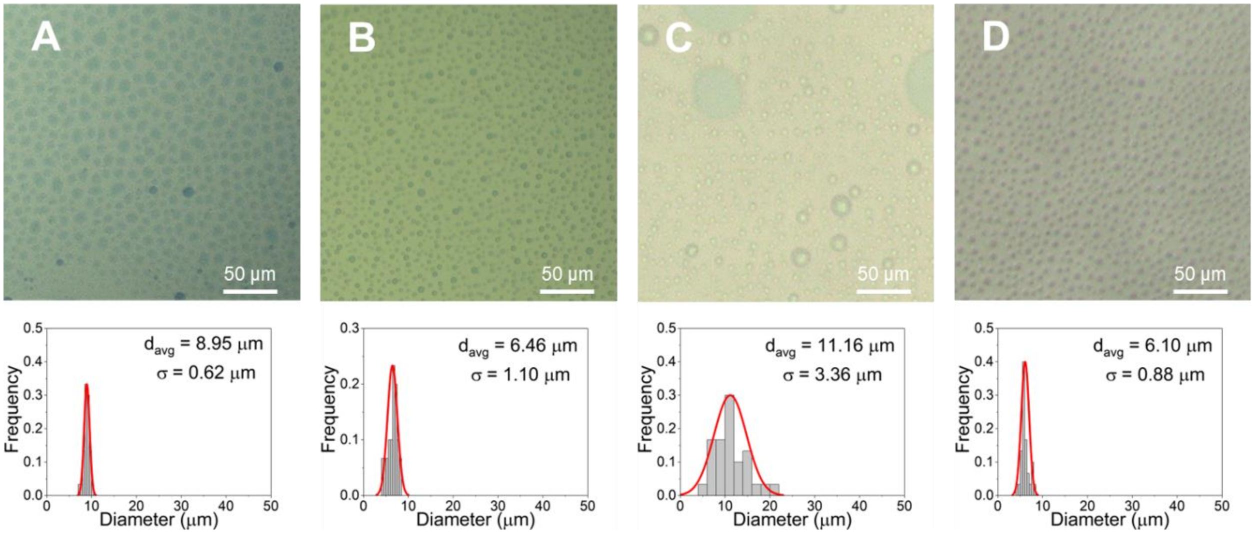

Fig. S10. Microscale colloidal phase behavior and size distributions at 10 wt.% IL concentration and $\boldsymbol{T_{sep}} = 70$ °C. **A** PTFA. **B** PDMBS. **C** NSal. **D** PSal. The PMR of all the ILs at this concentration is greater than 2.75, with the ILR phase (darker color) dispersed as discontinuous colloidal aggregates within a continuous WR phase (lighter color).

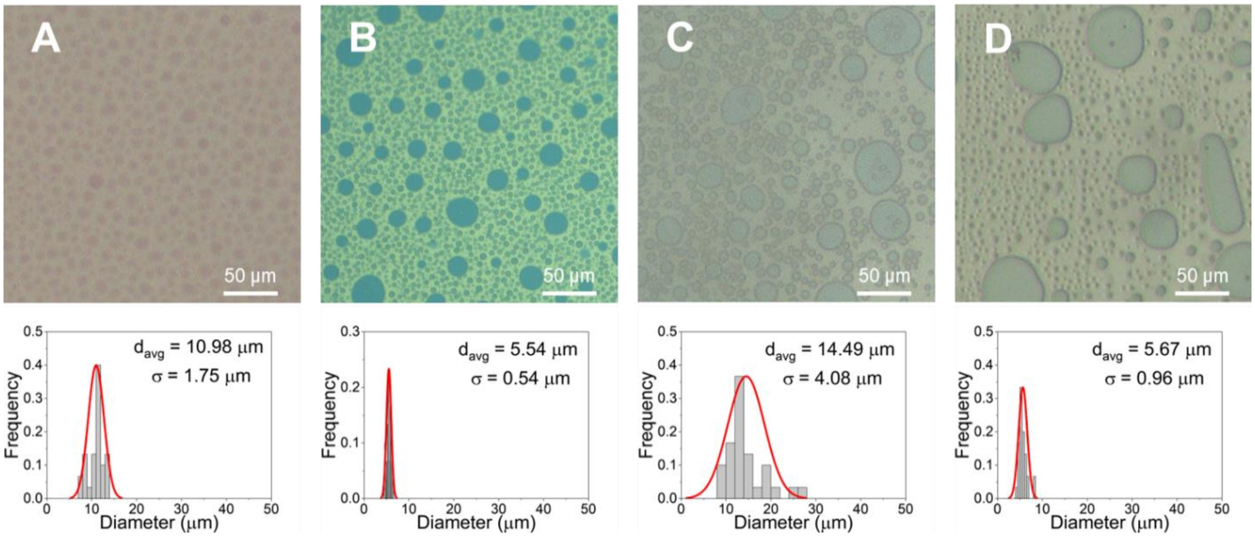

Fig. S11. Microscale colloidal phase behavior and colloidal size distributions at 20 wt.% IL concentration and $\boldsymbol{T_{sep}} = 70$ °C. **A** PTFA. **B** PDMBS. **C** NSal. **D** PSal. The PMR of all the ILs at this concentration is greater than 2.75, with the ILR phase (darker color) dispersed as discontinuous colloidal aggregates within a continuous WR phase (lighter color).

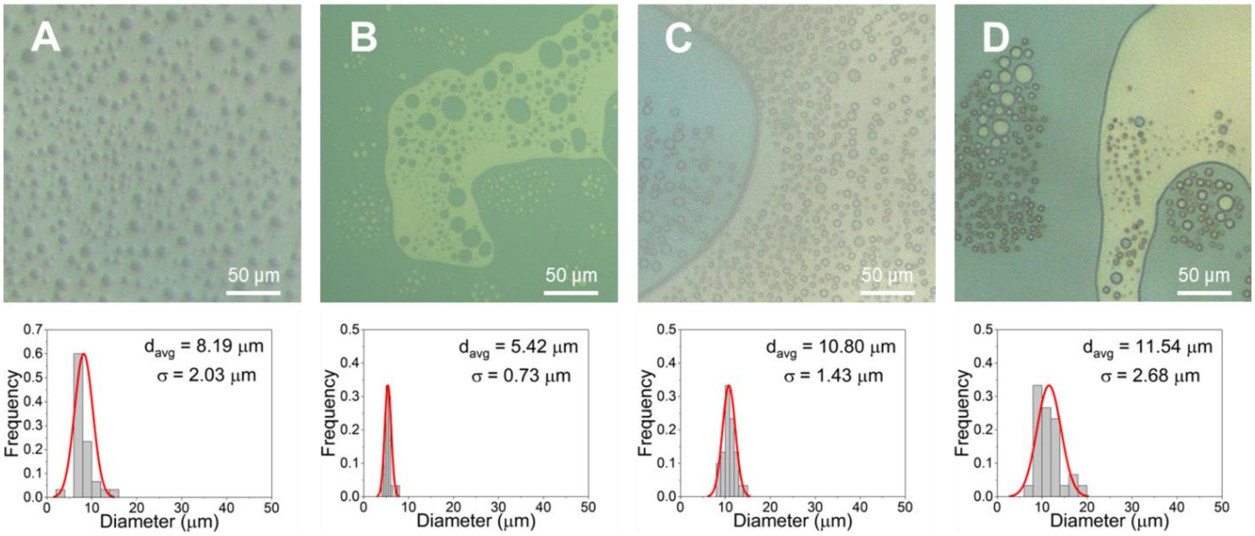

Fig. S12. Microscale colloidal phase behavior and colloidal size distributions at 30 wt.% IL concentration and $\boldsymbol{T_{sep}} =$ 70 °C. **A** PTFA. **B** PDMBS. **C** NSal. **D** PSal. The PMR of all the ILs at this concentration is less than or equal to 2.75. All ILs except PTFA form discontinuous WR and ILR phases. Size distributions correspond to the phase of the greatest population, with A to C showing the ILR phase, and D showing the WR phase.

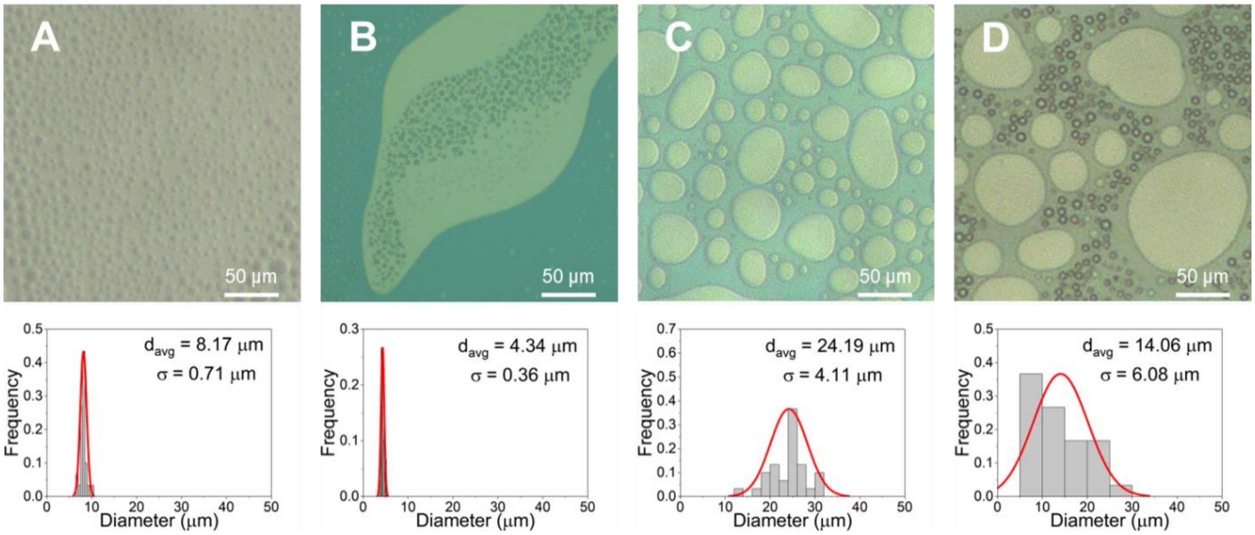

Fig. S13. Microscale colloidal phase behavior and colloidal size distributions at 40 wt.% IL concentration and $\boldsymbol{T_{sep}} =$ 70 °C. **A** PTFA. **B** PDMBS. **C** NSal. **D** PSal. The PMR of PDMBS is within the 2.75-0.75 PMR regime, hence both phases form discontinuous phasic colloids with the ILR dominating the distribution. The PMR of NSal and PSal is below 0.75, hence the WR phase (lighter color) forms discontinuous colloidal aggregates within a continuous ILR phase (darker color). Although PTFA is within the 2.75-0.75 PMR regime, the WR phase does not form discontinuous colloidal aggregates. Size distributions correspond to the phase of the greatest population, with A to B showing the ILR phase, and C to D showing the WR phase.

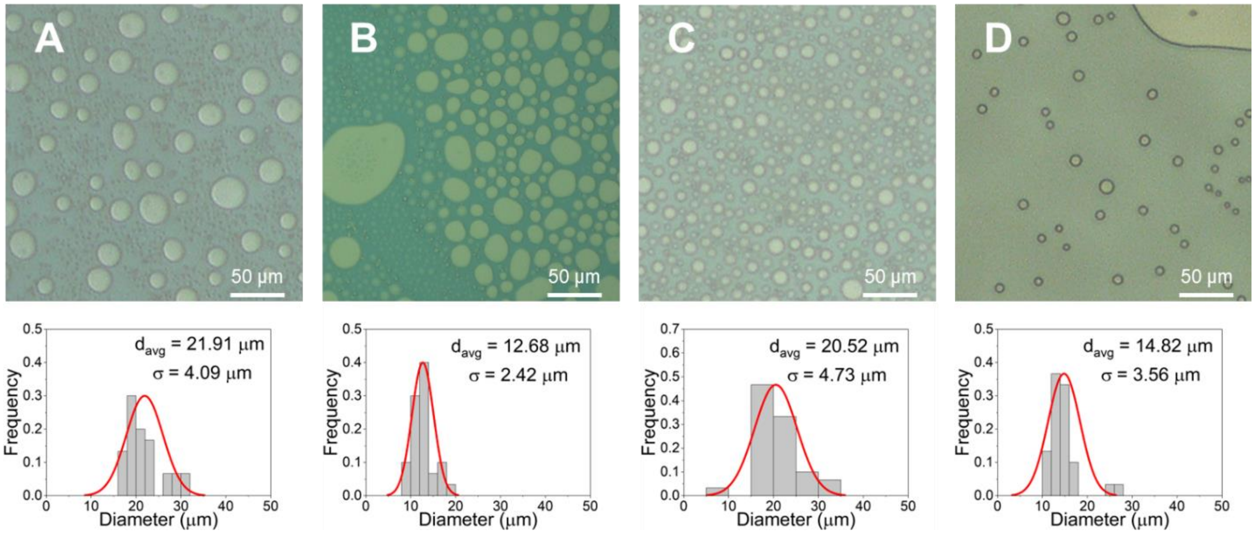

Fig. S14. Microscale colloidal phase behavior and colloidal size distributions at 50 wt.% IL concentration and $\boldsymbol{T_{sep}} =$ 70 °C. **A** PTFA. **B** PDMBS. **C** NSal. **D** PSal. The PMR of all the ILs at this concentration is less than 0.75, and hence the WR forms (lighter color) colloidal aggregates within a continuous ILR phase (darker color).

Table S2. Summary of mean colloidal sizes ($\boldsymbol{d_{avg}}$) and distribution standard deviations ($\boldsymbol{\sigma}$) shown in Fig. S11 to Fig. S15.

IL Concentration (wt.%)	PTFA		PDMBS		NSal		PSal	
	d_{avg} (µm)	σ (µm)	d_{avg} (µm)	σ (µm)	d_{avg}(µm)	σ (µm)	d_{avg} (µm)	σ (µm)
10	8.95	0.62	6.46	1.10	11.16	3.36	6.10	0.88
20	10.98	1.75	5.54	0.54	14.49	4.08	5.67	0.96
30	8.19	2.03	5.42	0.73	10.80	1.43	11.54	2.68
40	8.17	0.71	4.34	0.36	24.19	4.11	14.06	6.08
50	21.91	4.09	12.68	2.42	20.52	4.73	14.82	3.56

References

1. Kell, G.S., *Density, thermal expansivity, and compressibility of liquid water from 0. deg. to 150. deg.. Correlations and tables for atmospheric pressure and saturation reviewed and expressed on 1968 temperature scale.* Journal of Chemical and Engineering data, 1975. **20**(1): p. 97–105.
2. Kamio, E., et al., *Fundamental investigation of osmolality, thermo-responsive phase diagram, and water-drawing ability of ionic-liquid-based draw solution for forward osmosis membrane process.* Journal of membrane science, 2019. **570**: p. 93–102.
3. Haddad, A.Z., et al., *Solar desalination using thermally responsive ionic liquids regenerated with a photonic heater.* Environmental science & technology, 2021. **55**(5): p. 3260–3269.
4. Raman, C. and S. Ramaseshan. *Diffraction of light by transparent spheres and spheroids: the Fresnel patterns*. in *Proceedings of the Indian Academy of Sciences-Section A*. 1949. Springer.
